# Individual and Household Risk Factors for Symptomatic Cholera Infection: A Systematic Review and Meta-analysis

**DOI:** 10.1093/infdis/jiy444

**Published:** 2018-08-18

**Authors:** Aaron Richterman, Duarxy Rodcnel Sainvilien, Lauren Eberly, Louise C Ivers

**Affiliations:** 1 Department of Medicine, Brigham and Women’s Hospital, Boston, Massachusetts; 2 Department of Medicine, Hôpital Universitaire de Mirebalais, Haiti; 3 Center for Global Health, Massachusetts General Hospital; 4 Department of Global Health and Social Medicine, Harvard Medical School, Boston, Massachusetts

**Keywords:** cholera, *V. cholerae*, risk factors, predictor, neglected tropical diseases

## Abstract

**Background:**

Cholera has caused 7 global pandemics, including the current one which has been ongoing since 1961. A systematic review of risk factors for symptomatic cholera infection has not been previously published.

**Methods:**

In accordance with PRISMA (Preferred Reporting Items for Systematic Reviews and Meta-Analyses) guidelines, we performed a systematic review and meta-analysis of individual and household risk factors for symptomatic cholera infection.

**Results:**

We identified 110 studies eligible for inclusion in qualitative synthesis. Factors associated with symptomatic cholera that were eligible for meta-analysis included education less than secondary level (summary odds ratio [SOR], 2.64; 95% confidence interval [CI], 1.41–4.92; *I*^*2*^ = 8%), unimproved water source (SOR, 3.48; 95% CI, 2.18–5.54; *I*^*2*^ = 77%), open container water storage (SOR, 2.03; 95% CI, 1.09–3.76; *I*^*2*^ = 62%), consumption of food outside the home (SOR, 2.76; 95% CI, 1.62–4.69; *I*^*2*^ = 64%), household contact with cholera (SOR, 2.91; 95% CI, 1.62–5.25; *I*^*2*^ = 89%), water treatment (SOR, 0.37; 95% CI, .21–.63; *I*^*2*^ = 74%), and handwashing (SOR, 0.29; 95% CI, .20–.43; *I*^*2*^ = 37%). Other notable associations with symptomatic infection included income/wealth, blood group, gastric acidity, infant breastfeeding status, and human immunodeficiency virus infection.

**Conclusions:**

We identified potential risk factors for symptomatic cholera infection including environmental characteristics, socioeconomic factors, and intrinsic patient factors. Ultimately, a combination of interventional approaches targeting various groups with risk-adapted intensities may prove to be the optimal strategy for cholera control.

Cholera, the acute watery diarrheal illness caused by toxigenic *Vibrio cholerae*, is endemic to the Indian subcontinent and has caused 7 recorded global pandemics [1]. The seventh pandemic has been ongoing since 1961 and has extended throughout Asia into Africa, Europe, and the Americas. In 1855, John Snow first identified contaminated water as an individual risk factor for symptomatic cholera [2, 3]. Since then, many studies have explored other potential risk factors in a variety of settings, and yet overall rates of cholera have not measurably decreased, with the 172454 cholera cases reported in 2015 representing a fraction of the estimated 1.3–4.0 million annual cases worldwide [4, 5]. As the oral cholera vaccine continues to be more widely used as a targeted tool for cholera control [6], it will be important to consider if the evidence that exists for groups and individuals at high risk for cholera is being used to full capacity to inform implementation of this (and other) interventions. In this systematic review and meta-analysis, we thus sought to identify and summarize all known individual and household risk factors for symptomatic cholera.

## METHODS

We conducted this study in accordance with PRISMA (Preferred Reporting Items for Systematic Reviews and Meta-Analyses) guidelines, which provide evidence-based recommendations for conducting and reporting systematic reviews and meta-analyses [7].

### Eligibility Criteria

We searched for peer-reviewed articles assessing individual or household risk factors for symptomatic cholera infection, either with microbiologic confirmation or as defined by the World Health Organization (WHO) case definition of cholera: a patient ≥5 years of age with acute watery diarrhea, with or without vomiting, in an area with a known cholera epidemic [8]. We considered studies focused on the following to be beyond the scope of this review: subclinical cholera infection; neighborhood-, district-, or national-level risk factors for cholera; risk factors for cholera severity or mortality; and studies assessing protection from cholera provided by oral cholera vaccine, the subject of a recent meta-analysis [6].

### Search Strategy

We searched PubMed, Embase, and the Cochrane Library using the following terms: (“cholera” OR “*Vibrio cholerae*”) AND (“risk” or “predict” or “risk factor”). We manually reviewed reference lists of related reviews and all included articles.

### Study Selection and Data Collection

After elimination of duplicate records, 2 reviewers independently screened abstracts of all records for full-text review. After screening, 2 reviewers independently applied eligibility criteria to each full-text article and proceeded to data extraction for eligible studies using a standardized form created for the study (Supplementary Table 1). Disagreements were settled by discussion among all authors.

### Assessment of Bias

We assessed risk of bias within nonrandomized studies using the Newcastle–Ottawa Scale [9]. There were no randomized studies included in this review. We generated funnel plots and visually inspected for publication bias.

### Data Analysis

All included studies were summarized in a qualitative synthesis. We considered a risk factor for meta-analysis if it was assessed in an analogous way by >1 study, and included studies that reported an effect measure, implemented a multivariable model, and used community or household controls. We included effect measures generated in multivariable models when available. We generated summary odds ratios (SORs) using random effects models and a generic inverse variance approach to allow for inclusion of odds ratios controlled for measured confounders. We assessed heterogeneity with the Cochran *Q* test and the *I*^*2*^ statistic.

We performed a prespecified sensitivity analysis for risk factors included in meta-analysis by assessing for a subgroup difference between studies with and without microbiologic confirmation of cholera infection. We also evaluated for a subgroup difference between studies that took place in endemic settings compared to epidemic settings.

Data were analyzed using Review Manager (RevMan) version 5.3 (The Cochrane Collaboration, Copenhagen: Nordic Cochrane Center).

## RESULTS

### Study Selection

**Figure 1.**
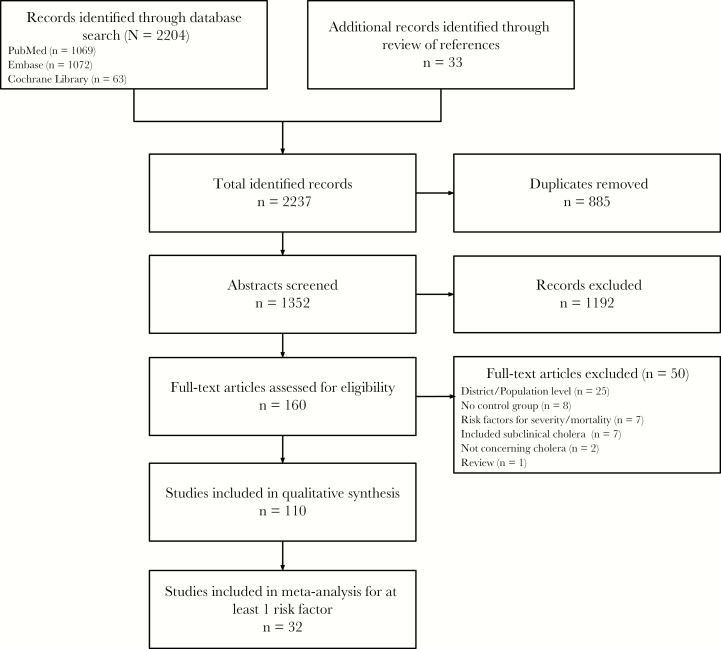
Flow diagram summarizing literature search.

The database search was performed on 18 January 2018 and is summarized in Figure 1. After removal of 885 duplicates, 1352 abstracts were screened, yielding 160 full-text articles for review. After applying inclusion and exclusion criteria, there were 110 remaining articles for inclusion in the qualitative synthesis (Supplementary Table 2) [10–119]. Of these, 32 studies ultimately qualified to be included in meta-analysis for at least 1 potential risk factor for cholera.

### Demographics

The majority of studies were both age- and sex-matched analyses, but a subset assessed risk for cholera based on these characteristics. Fifteen studies assessed differential risk by age [13, 26, 32, 33, 35, 47, 53, 70, 82, 85, 97, 101, 104, 110, 118], 10 of which implemented a multivariable model [26, 32, 33, 35, 82, 97, 101, 104, 110, 118]. Age was measured heterogeneously and findings were mixed. Four studies found that younger patients were at higher risk for cholera [26, 35, 101, 110], and 1 study found that older children had higher risk than children <1 year old [32]. Other studies, however, found older people to be at higher risk [33, 97], or no difference in risk based on age [82, 104, 118].

The role of sex was evaluated in 15 studies [13, 26, 32, 33, 35, 37, 43, 70, 72, 73, 90, 101, 104, 116, 118], of which 11 implemented a multivariable model [32, 33, 35, 37, 72, 73, 90, 101, 104, 116, 118]. Seven of these studies met criteria for meta-analysis (Supplementary Figure 1) [35, 72, 73, 90, 101, 116, 118]. The SOR for risk of cholera for females was 1.22 (95% confidence interval [CI], .71–2.12), with *I*^*2*^ = 80% (*Q* test *P* < .0001).

### Socioeconomic Factors

Seven studies directly assessed the relationship between income and risk of cholera [13, 18, 32, 33, 39, 43, 116]. Three of these implemented multivariable models: 2 found that people with cholera were more likely to come from lower-income households compared to those with noncholera diarrhea [32, 33], and 1 found no difference in cholera risk by income [116].

Seven studies considered socioeconomic status as measured by asset ownership or composite wealth index [17, 41, 49, 54, 70, 85, 90]. Four implemented a multivariable analysis [41, 49, 54, 90], of which 3 found lower socioeconomic status to be independently associated with risk of cholera [49, 54, 90].

Eight studies evaluated household building materials [10, 32, 33, 41, 43, 73, 101, 110]. Seven of these implemented multivariable models [10, 32, 33, 41, 73, 101, 110], 5 of which found that higher-quality housing was associated with lower risk of cholera [10, 32, 33, 101, 110]. One study found that risk of cholera independently increased with population density surrounding a household [101].

**Figure 2.**
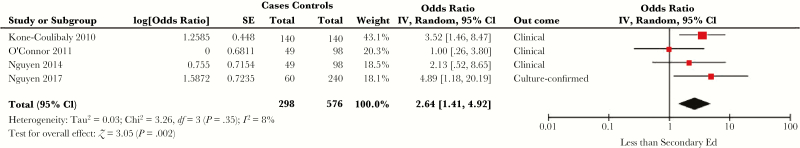
Forest plot of studies included in meta-analysis assessing whether an individual having less than secondary education was associated with symptomatic cholera. The summary odds ratio was calculated using random effects models. Heterogeneity is described using the Cochran *Q* test and the *I*^2^ statistic. Abbreviations: CI, confidence interval; *df*, degrees of freedom; IV, inverse variance; SE, standard error.

Fourteen studies explored the role of education [18, 26, 32, 33, 41, 43, 56, 65, 73, 82, 83, 85, 86, 101], of which 11 implemented a multivariable model [26, 32, 33, 41, 56, 65, 73, 82, 83, 85, 86]. Four studies looked specifically at whether an individual had some secondary education and met criteria for meta-analysis (Figure 2) [65, 82, 83, 86]. The SOR for cholera with less than some secondary education was 2.64 (95% CI, 1.41–4.92), with *I*^*2*^ = 8% (*Q* test *P* = .35).

There were mixed findings by 8 studies assessing the relationship between number of household members and risk of cholera [18, 33, 49, 54, 73, 101, 116, 118]. Of the studies that implemented multivariable models, 3 found higher risk with more members [33, 54, 118], 1 found lower risk with more members [49], and 3 found no association [73, 101, 116].

Four studies found no relationship between cholera and electricity in the household [70, 73, 86, 118], 3 found no relationship with literacy [26, 41, 116], and 2 found no relationship with household size [17, 43].

### Water

**Figure 3.**
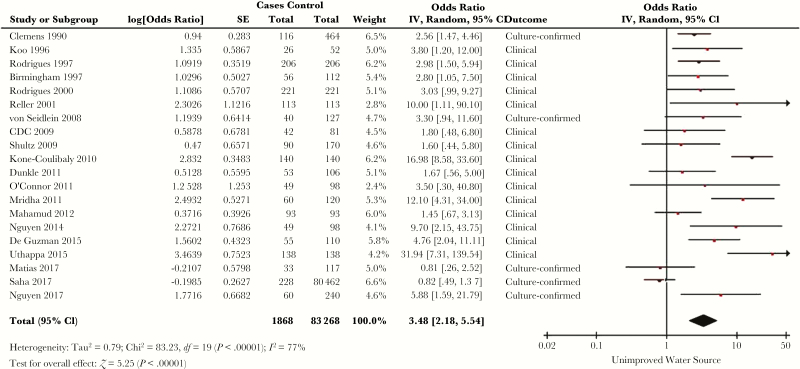
Forest plot of studies included in meta-analysis assessing whether exposure to an unimproved water source was associated with symptomatic cholera. The summary odds ratio was calculated using random effects models. Heterogeneity is described using the Cochran *Q* test and the *I*^2^ statistic. Abbreviations: CI, confidence interval; *df*, degrees of freedom; IV, inverse variance; SE, standard error.

Fifty-six studies evaluated risk of cholera based on source of water, with many identifying a specific culprit [17–19, 21, 22, 25–27, 30, 32, 33, 36, 37, 41–43, 47, 50, 59–61, 63, 65, 66, 68, 70, 72–74, 76, 78, 79, 81–83, 85–88, 91, 92, 94, 95, 97, 101–108, 110, 113, 116, 118]. Thirty-one studies implemented a multivariable model [19, 26, 27, 30, 32, 33, 36, 37, 41, 42, 60, 65, 66, 68, 72, 73, 78, 79, 82, 83, 86, 91, 92, 94, 95, 101, 104–106, 116, 118]. Of these, 20 measured the water source in a way that could be classified as improved (piped household, protected well or spring, or collected rainwater) or unimproved, and met criteria for meta-analysis (Figure 3) [19, 27, 30, 37, 41, 65, 66, 72, 73, 78, 82, 83, 86, 91, 94, 95, 101, 106, 116, 118]. The SOR for risk of cholera with an unimproved water source was 3.48 (95% CI, 2.18–5.54), with *I*^*2*^ = 77% (*Q* test *P* < .00001). Of the 11 studies excluded from meta-analysis, 1 found a significant association but did not report an effect measure [92], 6 used hospital controls [32, 33, 60, 68, 104, 105], and 4 did not define water source in way comparable to the others [26, 42, 79, 110].

**Figure 4.**
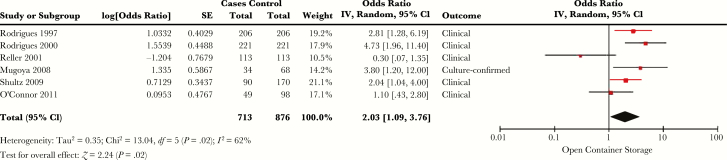
Forest plot of studies included in meta-analysis assessing whether water storage with an open container or bucket was associated with symptomatic cholera. The summary odds ratio was calculated using random effects models. Heterogeneity is described using the Cochran *Q* test and the *I*^*2*^ statistic. Abbreviations: CI, confidence interval; *df*, degrees of freedom; IV, inverse variance; SE, standard error.

Methods of water storage were assessed by 17 studies [17, 19, 21, 25, 35, 50, 54, 64, 79, 86, 88, 91, 92, 94, 95, 106, 112]. Of the 12 that included multivariable analyses [19, 35, 54, 64, 79, 86, 91, 92, 94, 95, 106, 112], 6 looked at storing water in a bucket or open container and were eligible for meta-analysis (Figure 4) [79, 86, 91, 94, 95, 106]. The SOR for risk of cholera with storing water in a bucket or open container was 2.03 (95% CI, 1.09–3.76), with *I*^*2*^ = 62% (*Q* test *P* = .02).

**Figure 5.**
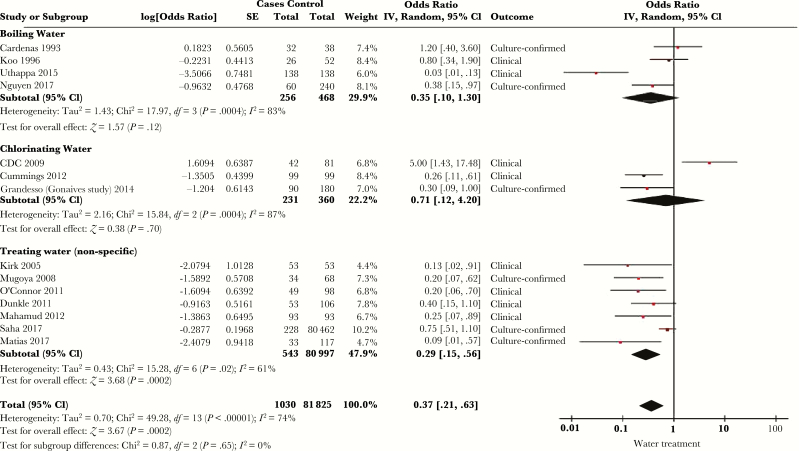
Forest plot of studies included in meta-analysis assessing whether water treatment was associated with symptomatic cholera. Studies were stratified by whether they assessed chlorination, boiling, or nonspecific report of water treatment. The summary odds ratio was calculated using random effects models. Heterogeneity is described using the Cochran *Q* test and the *I*^*2*^ statistic. Abbreviations: CI, confidence interval; *df*, degrees of freedom; IV, inverse variance; SE, standard error.

Thirty-nine studies considered the relationship between risk of cholera and household water treatment, typically in the form of either chlorination or boiling [10, 13, 17, 20, 26, 27, 32–35, 37, 39–41, 47, 49, 50, 56, 64, 66, 72, 73, 79, 80, 82, 86, 88, 91, 92, 96, 101–103, 108, 112, 114–116, 119]. Twenty-seven of these studies used a multivariable model [10, 26, 27, 32, 33, 35, 37, 40, 41, 49, 56, 64, 66, 72, 73, 79, 80, 82, 86, 91, 92, 96, 101, 112, 115, 116, 119], 15 of which met criteria for meta-analysis after stratification into type of water treatment (chlorination, boiling water, or nonspecific) (Figure 5) [26, 27, 35, 41, 49, 64, 66, 72, 73, 79, 82, 86, 101, 115, 116]. The overall SOR for cholera with water treatment was 0.37 (95% CI, .21–.63), with *I*^*2*^ = 74% (*Q* test *P* < .00001). There was no significant difference between the subgroups (*P* = .65). Of the 12 multivariable analyses excluded from meta-analysis, 8 did not report an effect measure (3 found a significant relationship between water treatment and cholera, and 5 did not) [37, 40, 80, 91, 92, 96, 112, 119]. Three studies directly assessed chlorine concentration in household water; none found an association with cholera risk [25, 49, 86].

Distance to a water source was assessed in 5 studies which implemented multivariable models [10, 27, 30, 32, 33]. Three studies found that increased distance from a water source was associated with cholera risk [10, 32, 33], 1 found the opposite [27], and 1 study found that proximity to a contaminated river was associated with increased cholera risk [30].

Two studies found no relationship between availability of water and risk of cholera [26, 85].

### Food

Reported exposure to a specific food was commonly assessed. In particular, the role of seafood was evaluated in 23 studies [10, 15, 17, 19, 21, 23, 42, 43, 46, 47, 56, 60, 63, 69, 70, 74, 83, 95, 109, 112, 113, 118, 119]. Implicated seafood items in multivariable analyses included raw or partially cooked seafood [56, 119], crabs or shellfish [83, 95], dried fish [10], and seafood salad [42]. Thirty studies looked at other types of food exposures [13, 17, 19, 21, 40, 42, 47, 54, 55, 60, 61, 66, 69, 70, 72, 75, 80, 82, 83, 86, 87, 94, 95, 97, 105, 109, 111, 112, 114, 118]. Notable among these were 6 multivariable analyses with mixed results on vegetable exposure; some found a significant relationship between cholera and raw vegetable exposure [40, 75, 80], others did not [83, 105], and 1 study found steamed vegetables to be associated with reduced risk of cholera [82].

Twenty-four studies assessed risk of cholera with exposure to food from street vendors or outside the home [26, 35, 41, 49, 56, 57, 66, 68, 70, 72, 73, 78, 79, 83–85, 88, 91, 92, 103, 109, 115, 118, 119]. Seventeen studies implemented multivariable models [35, 41, 49, 56, 66, 68, 72, 73, 78, 79, 83, 91, 92, 109, 115, 118, 119], and 12 could be summarized using meta-analysis (Supplementary Figure 2) [35, 41, 49, 66, 72, 73, 78, 79, 83, 109, 115, 118]. The SOR of cholera risk with exposure to street vendor food or food from outside the home was 2.76 (95% CI, 1.62–4.69), with *I*^*2*^ = 64% (*Q* test *P* = .0008). Of the 5 controlled studies not eligible for meta-analysis, 3 did not report an effect measure [91, 92, 119].

There were mixed results when looking at cholera risk based on hot meal preparation or exposure to leftover food [27, 35, 41, 60, 65, 66, 75, 76, 80, 83, 88, 91, 92, 106, 109, 113, 118]. Of the 14 studies that used multivariable models, 4 found increased risk of cholera with a cold meal whereas 2 found no difference in risk [27, 35, 65, 83, 91, 118], and 3 found increased risk of cholera with leftover food whereas 2 found no difference in risk [41, 60, 66, 75, 80, 92, 106, 109].

The possible protective role of breastfeeding in the setting of cholera was first noted in 1979, when bottle-fed children in a matched case-control study had significantly higher risk of cholera [50]. This relationship has been further explored [30, 32, 43, 89], including by 2 multivariable analyses that confirm the association between breastfeeding status and reduced risk of cholera [30, 32].

One study found that retinol deficiency was associated with a higher likelihood of developing symptomatic disease among people growing *V. cholerae* in their stool [53], and another found that prior retinol supplementation was associated with risk of cholera in children, hypothesized by the authors to reflect an underlying deficiency [32].

No studies evaluated risk of cholera based on access to food or food security, although one multivariable analysis found dietary diversity to be associated with a reduced risk of cholera [41]. No studies assessed nutritional status and risk of cholera.

### Latrines

Access to a flush toilet, latrine, or open defecation was included as a potential risk factor in 27 studies [10, 18, 20, 27, 32, 33, 35, 37, 39, 41, 43, 49, 54, 64, 73, 79, 82, 85–87, 90, 94, 97, 101–103, 107]. Of these, 18 implemented a multivariable model, with mixed results [10, 27, 32, 33, 35, 37, 41, 49, 54, 64, 73, 79, 82, 86, 90, 94, 97, 101]. Seven studies found no significant difference in cholera risk with access to a latrine [35, 37, 41, 49, 79, 86, 90], 2 found increased risk with latrines [54, 73], 2 found increased risk with open defecation whereas 1 found no significant difference [10, 27, 97], and 4 found decreased risk with a flush toilet while 2 found no difference [32, 33, 64, 82, 94, 101].

Six studies assessed whether sharing a latrine was a risk factor for cholera [40, 43, 70, 72, 106, 118]. Four of these were multivariable analyses [40, 72, 106, 118], of which 2 found that a communal latrine was associated with risk of cholera [72, 106].

### Cholera Contacts and Proximity to Other Cases

**Figure 6.**
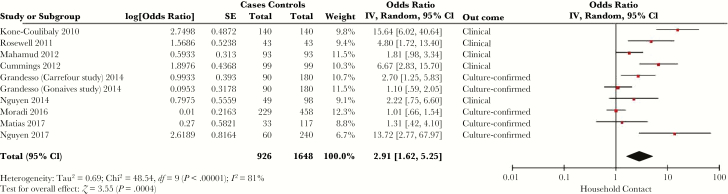
Forest plot of studies included in meta-analysis assessing whether presence of a household contact with cholera was associated with symptomatic cholera. The summary odds ratio was calculated using random effects models. Heterogeneity is described using the Cochran *Q* test and the *I*^*2*^ statistic. Abbreviations: CI, confidence interval; *df*, degrees of freedom; IV, inverse variance; SE, standard error.

The risk of cholera with a household contact with cholera was evaluated in 25 studies [20, 26, 32, 33, 35, 45, 47, 49, 51, 56, 57, 61, 63, 65, 72, 73, 75, 82, 83, 87, 96, 97, 105, 107, 110]. Fifteen studies of household contact included a multivariable model [26, 32, 33, 35, 49, 56, 65, 72, 73, 75, 82, 83, 96, 97, 105], of which 9 were eligible for meta-analysis (Figure 6) [35, 49, 65, 72, 73, 75, 82, 83, 96]. The SOR of cholera with a household contact with cholera was 2.91 (95% CI, 1.62–5.25), with *I*^*2*^ = 81% (*Q* test *P* < .00001). The 6 studies excluded from meta-analysis used hospital or clinic controls with either noncholera diarrhea or without diarrhea [26, 32, 33, 56, 97, 105], and all reported an association between cholera and a household contact with cholera.

Two studies looked specifically at sharing a latrine with a person with cholera; 1 found a significantly increased risk and 1 found no significant association [49, 105].

Two studies found that individuals living in close proximity to other cases had the greatest risk of cholera [38, 71].

### Hygiene

**Figure 7.**
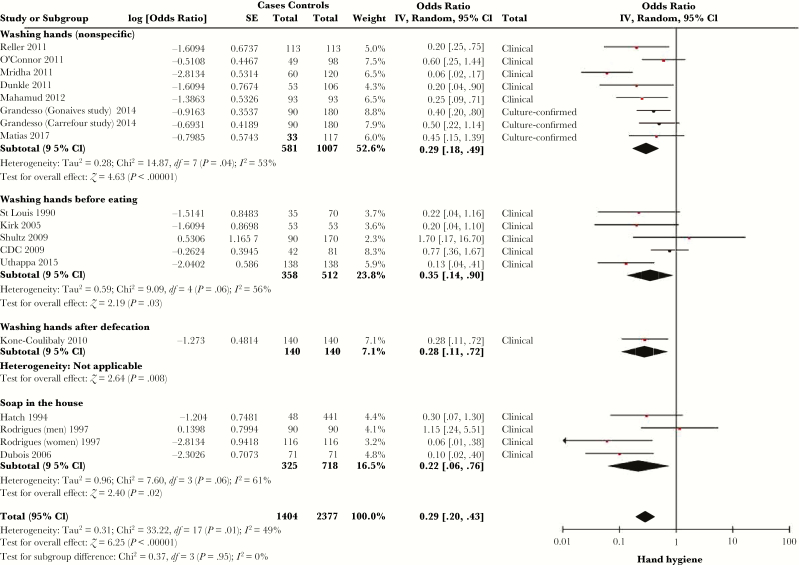
Forest plot of studies included in meta-analysis assessing whether handwashing was associated with symptomatic cholera. Studies were stratified by whether they assessed handwashing before eating, nonspecific handwashing, or presence of soap in the household. The summary odds ratio was calculated using random effects models. Heterogeneity is described using the Cochran *Q* test and the *I*^*2*^ statistic. Abbreviations: CI, confidence interval; *df*, degrees of freedom; IV, inverse variance; SE, standard error.

Hand hygiene was assessed in a number of ways, including handwashing before eating [20, 27, 54, 56, 60, 64, 79, 87, 94, 97, 102, 106, 109, 116], handwashing after defecation [20, 27, 35, 37, 40, 65, 79, 102, 106, 110], nonspecific handwashing [25, 39–41, 49, 61, 72, 73, 78, 86, 88, 91], and presence of soap in the home [54, 64, 72, 94, 97, 113, 119]. The vast majority of studies measuring handwashing relied on self-report. Sixteen studies, stratified by type of hand hygiene measured, met criteria for meta-analysis (Figure 7) [27, 40, 41, 49, 54, 64, 65, 72, 73, 78, 86, 91, 94, 106, 109, 116]. The SOR of cholera with hand hygiene was 0.29 (95% CI, .20–.43), with *I*^*2*^ = 49% (*Q* test *P* = .01), with no significant difference between the subgroups (*P* = .95). Three other studies that included handwashing after defecation in multivariable analyses but did not report effect measures found no significant association with cholera [35, 37, 79].

Of 4 studies evaluating the role of bathing in unsafe water, 2 found increased risk of cholera and 2 found no significant association [10, 19, 82, 109]. One multivariable analysis found no significant association between washing utensils with unsafe water and cholera risk [96].

### Attending a Gathering or Funeral

Fourteen included studies evaluated risk of cholera after attending a large gathering or funeral [10, 27, 51, 56, 63, 65, 79, 83, 96, 97, 105, 107, 112, 116]. Seven of these met criteria for meta-analysis (Supplementary Figure 3) [27, 65, 79, 83, 96, 105, 116]. The SOR of cholera after attending a large gathering or funeral was 2.42 (95% CI, 1.43–4.09), with *I*^*2*^ = 38% (*Q* test *P* = .14).

### Blood Type and Genetic Risk

Multiple studies found higher risk of symptomatic cholera among patients with blood group O [16, 28, 29, 48, 53], although those with blood group O appear to have lower risk of initial colonization by *V. cholerae* [52]. Among people with blood group A or B, the Lewis blood group Le(a^+^b^–^) was associated with symptomatic cholera in 1 study [14].

One study further explored the role of genetics in the risk of symptomatic cholera [62]. In a genome-wide association study, the authors noted evidence of natural selection in a Bengali population on genes in the NF-κB signaling pathway, which is implicated in proinflammatory response to *V. cholera* lipopolysaccharide. They went on to conduct a case-control study, finding that these genes were strongly associated with susceptibility to symptomatic cholera.

### Gastric Acidity

Decreased gastric acid levels have been implicated as a risk factor for cholera infection in several ways. In an experimental trial of cholera inoculation, buffering gastric acid led to a lower required infectious dose of *V. cholerae*, from 10^8^ organisms to 10^4^ [58]. Observational studies have found lower gastric acid levels in symptomatic cholera compared to noncholera diarrhea both during and after infection [44, 99, 117]. In one multivariable model, a positive *Helicobacter pylori* immunoglobulin G was associated with risk of cholera [31]. One study found higher risk of cholera among those who have had gastric surgery, although another did not [15, 69]. Antacid use was associated with cholera risk in 1 study, but not in 2 others where antacid use was low [69, 73, 111].

Four multivariable analyses assessed use of acidic additives to food. Three of these reported an effect measure and were candidates for meta-analysis (Supplementary Figure 4) [94, 95, 109]. The summary odds ratio of cholera with not using the acidic additive was 7.49 (95% CI, 2.10–26.68), with *I*^*2*^ = 72% (*Q* test *P* = .03).

### Human Immunodeficiency Virus

Two case-control studies, 1 using hospital controls and 1 with community controls, found an increased risk of cholera among people with human immunodeficiency virus (HIV) infection [104, 118].

### Prior Infection and Natural Immunity

A trial of experimental inoculation of volunteers with *V. cholerae* O1 demonstrated natural immunity to clinical infection when rechallenged 3 years later [67]. A case-control study in Bangladesh found a reduced risk of subsequent *V. cholerae* O1 infection with prior *V. cholerae* O1 infection, but no significant protection for *V. cholerae* O139 or cross-protection between serogroups [12]. In 2 studies, higher baseline vibriocidal titers were associated with protection from clinical infection of *V. cholerae* O1 in household contacts of cholera cases, but not for *V. cholerae* O139 [77, 100]. Another study found that vibriocidal titers did not predict risk of asymptomatic vs symptomatic illness among cholera household contacts with a positive stool culture for cholera, although lipopolysaccharide-specific antibodies were higher in symptomatic patients [53].

### Sensitivity Analysis

There were no significant subgroup differences for any of the variables included in meta-analysis when stratifying by whether studies used microbiologic confirmation of cholera cases or a clinical case definition, or whether the studies took place in an endemic setting or an epidemic setting.

### Bias

A summary of bias within studies can be found in Supplementary Table 3. Funnel plots were generated for all variables included in meta-analysis (Supplementary Figures 5–14). Visual inspection of the funnel plots suggested the presence of publication bias for the following variables: sex, improved water source, water storage, water treatment, street vendors, attendance of a gathering/funeral, and household contact with cholera.

## DISCUSSION

This systematic review of 110 studies and >22000 people with cholera identified factors associated with symptomatic infection related to water, sanitation, and hygiene (collectively referred to as WASH), other sources of exposure, socioeconomic status, and intrinsic patient characteristics.

Use of an unimproved water source conferred >3-fold increase in the odds of cholera in meta-analysis, highlighting the importance of a safe water source in cholera control. There was significant heterogeneity between the effects of water source on cholera risk among studies included in meta-analysis, probably reflecting differing degrees of contamination or exposure in different water sources. Use of an open container or bucket for water storage was also significantly associated with cholera, potentially through higher risk of water contamination. Water treatment, either by chlorination or boiling, was associated with lower risk of cholera. There was substantial heterogeneity among studies assessing water treatment, possibly reflecting differences in practice or reporting.

While access to adequate sanitation is likely to minimize risk of water supply contamination, use of latrines did not convincingly impact an individual’s risk of cholera, with the majority of well-controlled studies assessing individual latrine use finding no association with risk of cholera. Access to and use of hygiene measures, sanitation infrastructure, and sharing may impact cholera risk with latrine use, although none of this was definitively evident from the available information.

Handwashing, either before meals or generally, had a similar effect measure for decreased cholera risk as water treatment. This finding is consistent with a recent systematic review of the use of handwashing for prevention of diarrhea more generally [120]. Handwashing after defecation did not convincingly reduce cholera risk for the handwashing person, with 3 of 4 multivariable analyses assessing this variable finding no relationship but not reporting an effect measure. The majority of studies assessing handwashing relied on self-report rather than direct observation, introducing reporting and social desirability bias, such that the findings, while fitting with public health principles, should be interpreted with caution in terms of their specific usefulness to interrupt cholera epidemics. More specific microbiological studies may be needed to better understand the impact of handwashing on the interruption of cholera epidemics. Notably, despite the range of WASH factors that are associated with symptomatic cholera infection by such self-reported studies, a recent systematic review of WASH interventions found that it is not clear which interventions have impact in any given context [121].

Other non-water-related sources of exposure were also implicated in the risk of cholera. Food items, and seafood in particular, were associated with cholera in a number of studies. More generally, eating street food was associated with a 5-fold increase in the odds of cholera in meta-analysis, indicating that interventions with street vendors may be an important pathway to interrupt transmission during an outbreak, although current programmatic guidelines on how to address this are nonspecific [122]. Similarly, attending a large gathering or funeral during an outbreak was associated with cholera risk, and these gatherings may serve as a focus for ongoing cholera transmission. Additionally, having a household contact with cholera was associated with significantly increased risk of cholera in well-controlled studies. There was considerable heterogeneity between studies assessing both street vendor food and a household contact with cholera, indicating that these factors likely vary by setting. For example, local practices surrounding caring for an ill household contact, type of street food, and vendor practices may alter the magnitude of association between these factors and cholera risk.

Several socioeconomic factors were independently associated with risk of cholera. Both direct income and composite wealth were closely linked to cholera risk in all multivariable analyses assessing these variables. Education (in particular, less than secondary education) was associated with cholera risk. Taken together, these findings strongly point to the need to explore how poverty reduction, social support, and educational infrastructure could be used as interventions with a goal of cholera control.

Some intrinsic patient factors were also associated with cholera risk. Several genetic features, related to blood group O and genes in the NF-κB pathway, are associated with increased risk of symptomatic cholera, and appear to have contributed to natural selection in parts of Asia. Risk of cholera by age varied substantially between studies and may reflect differences in populations living in epidemic vs endemic areas, where natural immunity may play a role. Breastfeeding infants showed evidence of protection from cholera, probably as a result of passive immunity from breast milk and reduced likelihood of direct exposure. Reduced gastric acidity, from *H. pylori* infection or otherwise, decreased the required infectious dose of cholera, thus increasing the risk of symptomatic infection. Finally, HIV infection may increase risk of cholera, although there are only 2 studies assessing this association and both were at high risk for selection bias.

There was evidence of publication bias for some variables included in meta-analysis, including sex, water source, water treatment, water storage, street vendor food exposure, attending a large gathering or funeral, and household cholera contacts. The summary effect measures for these risk factors may thus be biased away from the null because negative associations are less likely to be reported or published. In some cases, studies that would have otherwise been eligible for meta-analysis did not report an effect measure. While generally there were relatively equal numbers of these studies reporting a null or significant association in one direction or the other, this may have introduced some bias into the resulting summary effect measures. We hypothesized that there might be differential risk associated with some variables when stratifying by whether or not cases were culture-confirmed, but this was not the case for any assessed risk factor included in meta-analysis. Regional differences may also contribute to heterogeneity in risk factors, although we did not find any differences based on epidemic or endemic setting in particular.

In sum, we identified potential risk factors for symptomatic cholera infection ranging from environmental characteristics directly impacting exposure risk, to socioeconomic factors such as wealth or education, to intrinsic patient factors such as specific genetic features or gastric acidity. Many of these potential risk factors can and have been intervened upon in cholera control efforts [122], although the relative efficacy and effectiveness of interventions targeting these factors as public health interventions remains a key gap in the literature [121]. Future studies should deepen our understanding of specific risk factors in water, sanitation, personal hygiene, and food hygiene, so that public health campaigns can go beyond generic advice often known as “key messages” and get deeper into specific recommendations that are known to reduce the risk of cholera. Additionally, our findings suggest that some high-risk groups (eg, people living with HIV) may warrant special attention during the cholera response. Ultimately, a combination of interventional approaches that target various groups with risk-adapted intensities may prove to be the most effective strategy.

## Supplementary Data

Supplementary materials are available at *The Journal of Infectious Diseases* online. Consisting of data provided by the authors to benefit the reader, the posted materials are not copyedited and are the sole responsibility of the authors, so questions or comments should be addressed to the corresponding author.

Supplementary Figure 1

Supplementary Figure 2

Supplementary Figure 3

Supplementary Figure 4

Supplementary Figure 5

Supplementary Figure 6

Supplementary Figure 7

Supplementary Figure 8

Supplementary Figure 9

Supplementary Figure 10

Supplementary Figure 11

Supplementary Figure 12

Supplementary Figure 13

Supplementary Figure 14

Supplementary Material

## Notes

***Financial support.*** This work was supported by grants from the Bill & Melinda Gates Foundation (grant number OPP1148213 to L. C. I.) and the National Institute of Allergy and Infectious Diseases (grant number R01AI099243 to L. C. I.).

***Supplement sponsorship.*** This work is part of a supplement sponsored by the Bill and Melinda Gates Foundation.

***Potential conflicts of interest.*** All authors: No potential conflicts of interest. All authors have submitted the ICMJE Form for Disclosure of Potential Conflicts of Interest. Conflicts that the editors consider relevant to the content of the manuscript have been disclosed.
