# Supplementary figures and images for: Individual and Household Risk Factors for Symptomatic Cholera Infection: A Systematic Review and Meta-analysis
Source: J Infect Dis. 2018 Aug 18;218(Suppl 3):S154–64. doi: 10.1093/infdis/jiy444 (PMC6188541; doi:10.1093/infdis/jiy444)

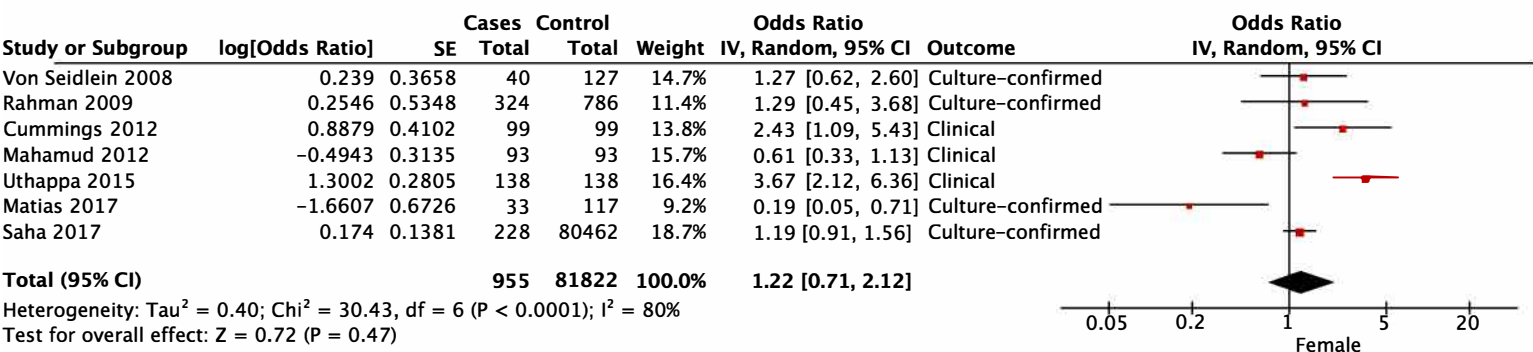

Supplement: Supplementary Figure 1 [file jiy444_suppl_supplementary_figure_1.pdf]

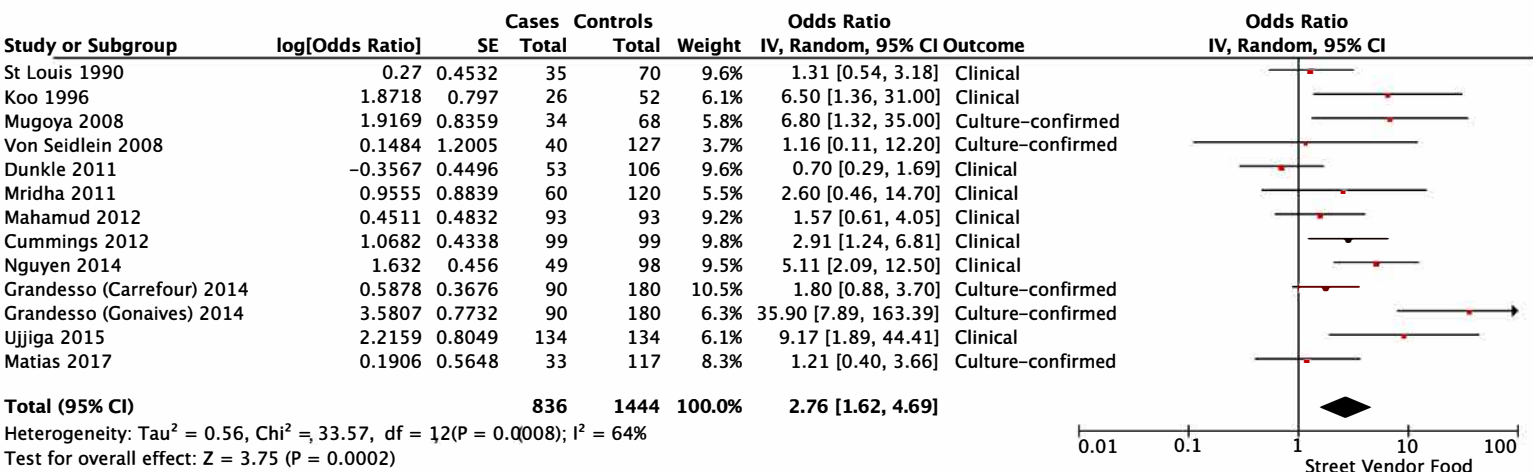

Supplement: Supplementary Figure 2 [file jiy444_suppl_supplementary_figure_2.pdf]

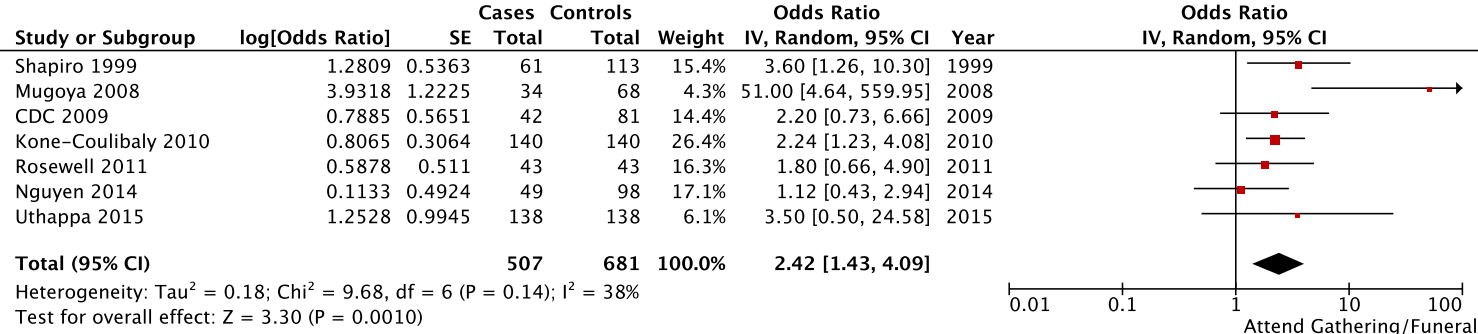

Supplement: Supplementary Figure 3 [file jiy444_suppl_supplementary_figure_3.pdf]

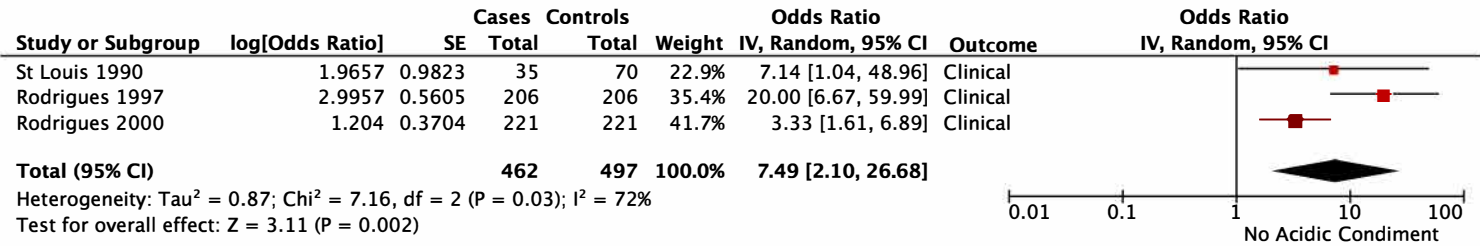

Supplement: Supplementary Figure 4 [file jiy444_suppl_supplementary_figure_4.pdf]

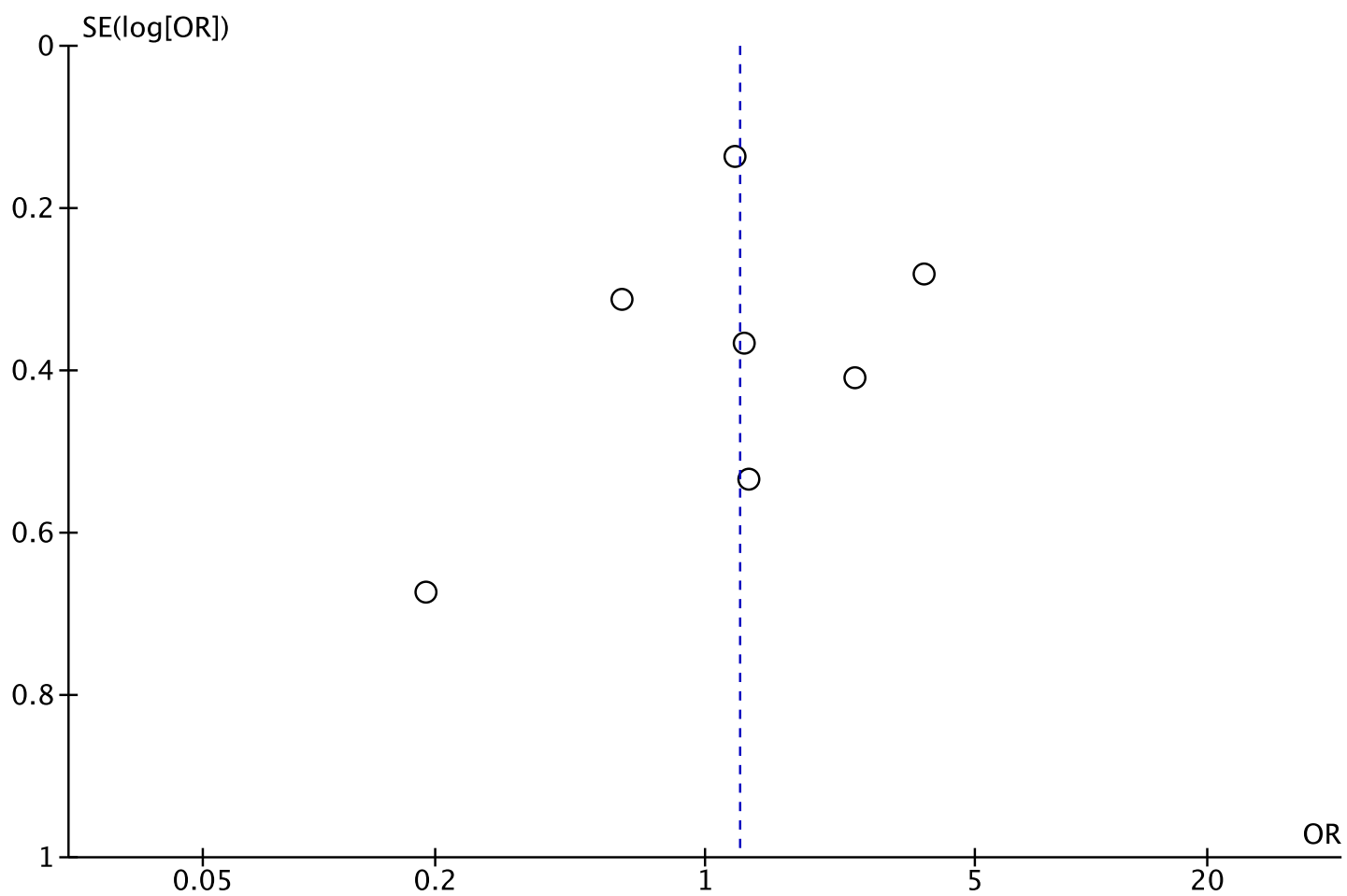

Supplement: Supplementary Figure 5 [file jiy444_suppl_supplementary_figure_5.pdf]

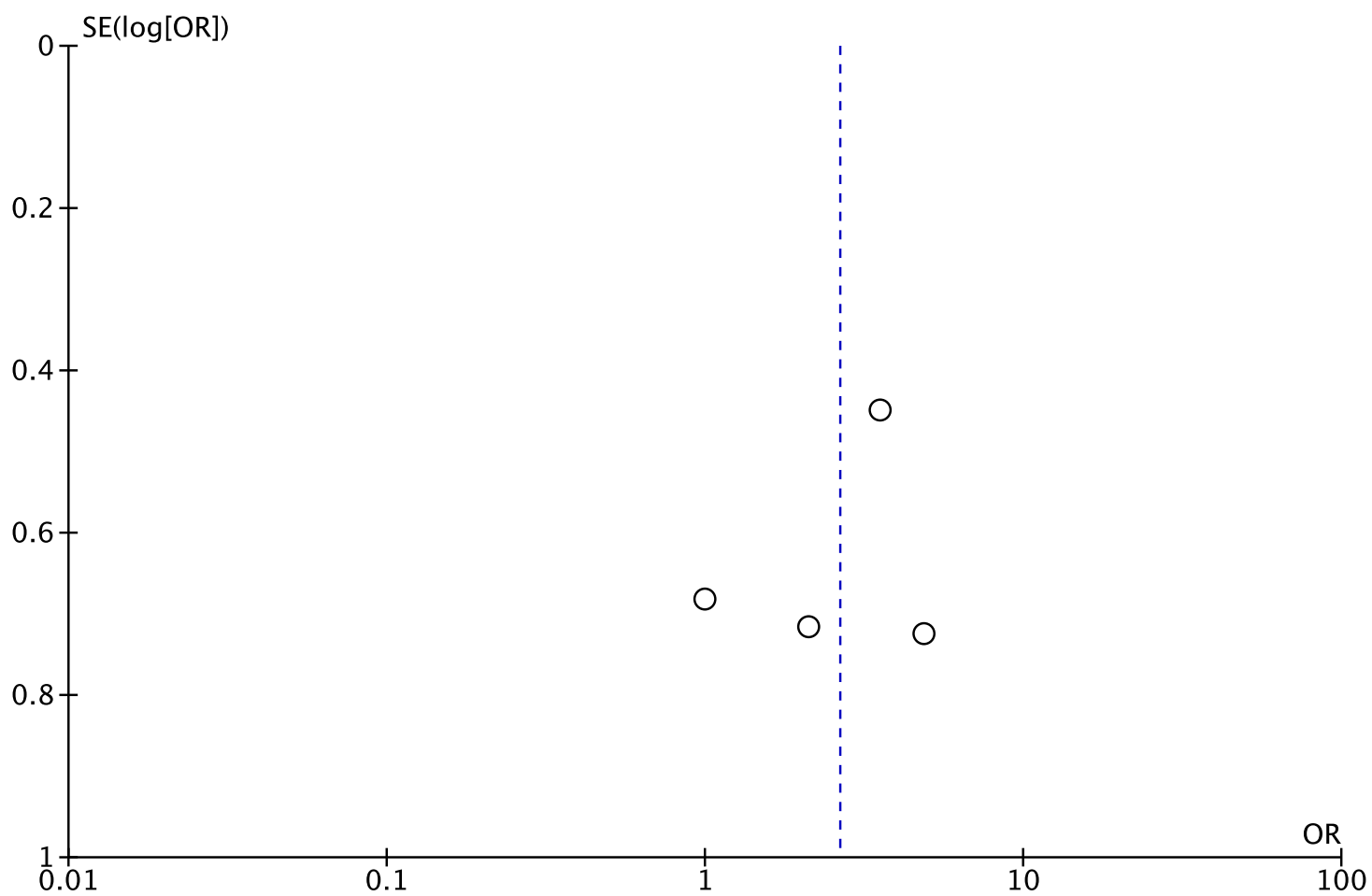

Supplement: Supplementary Figure 6 [file jiy444_suppl_supplementary_figure_6.pdf]

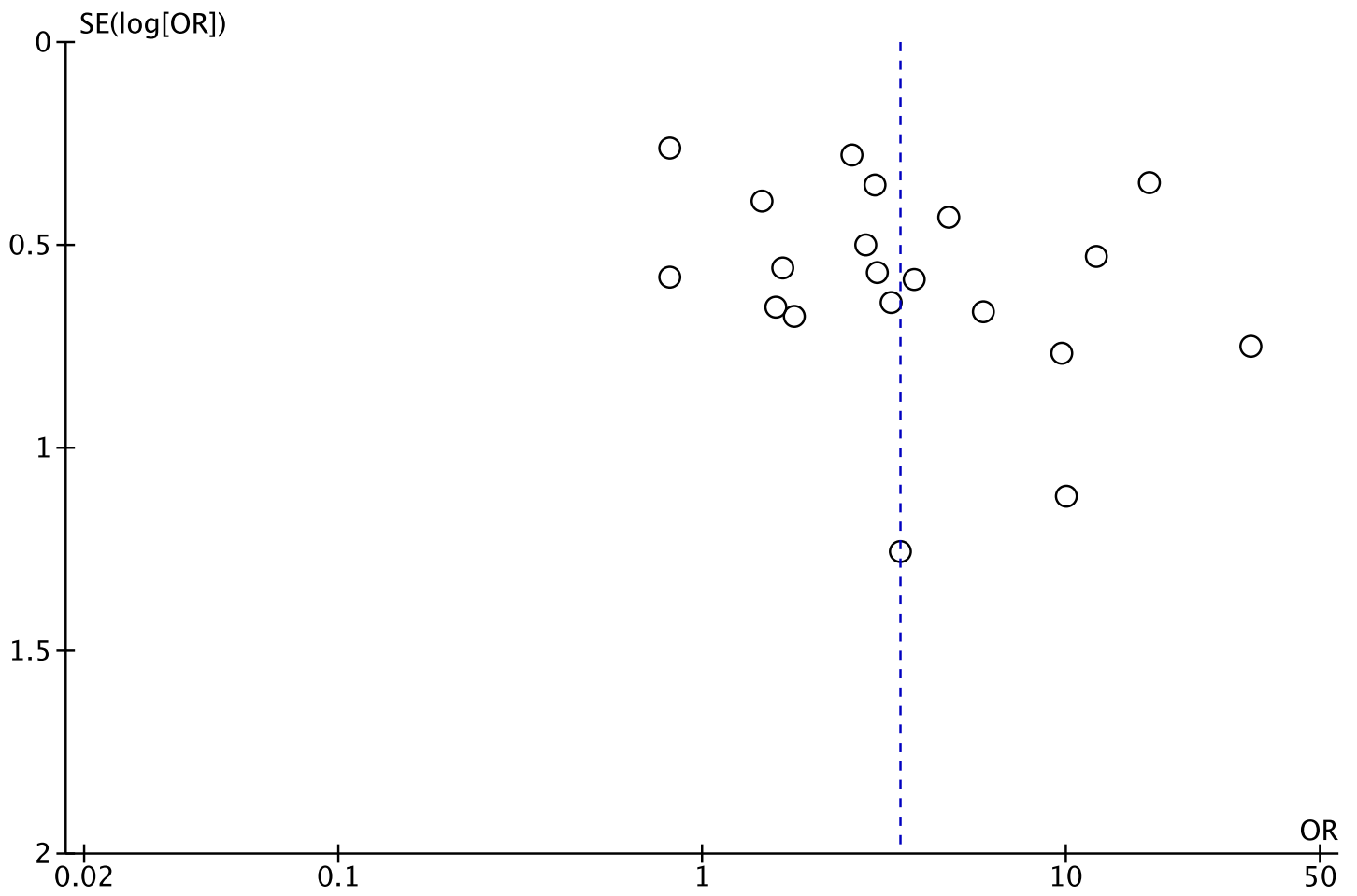

Supplement: Supplementary Figure 7 [file jiy444_suppl_supplementary_figure_7.pdf]

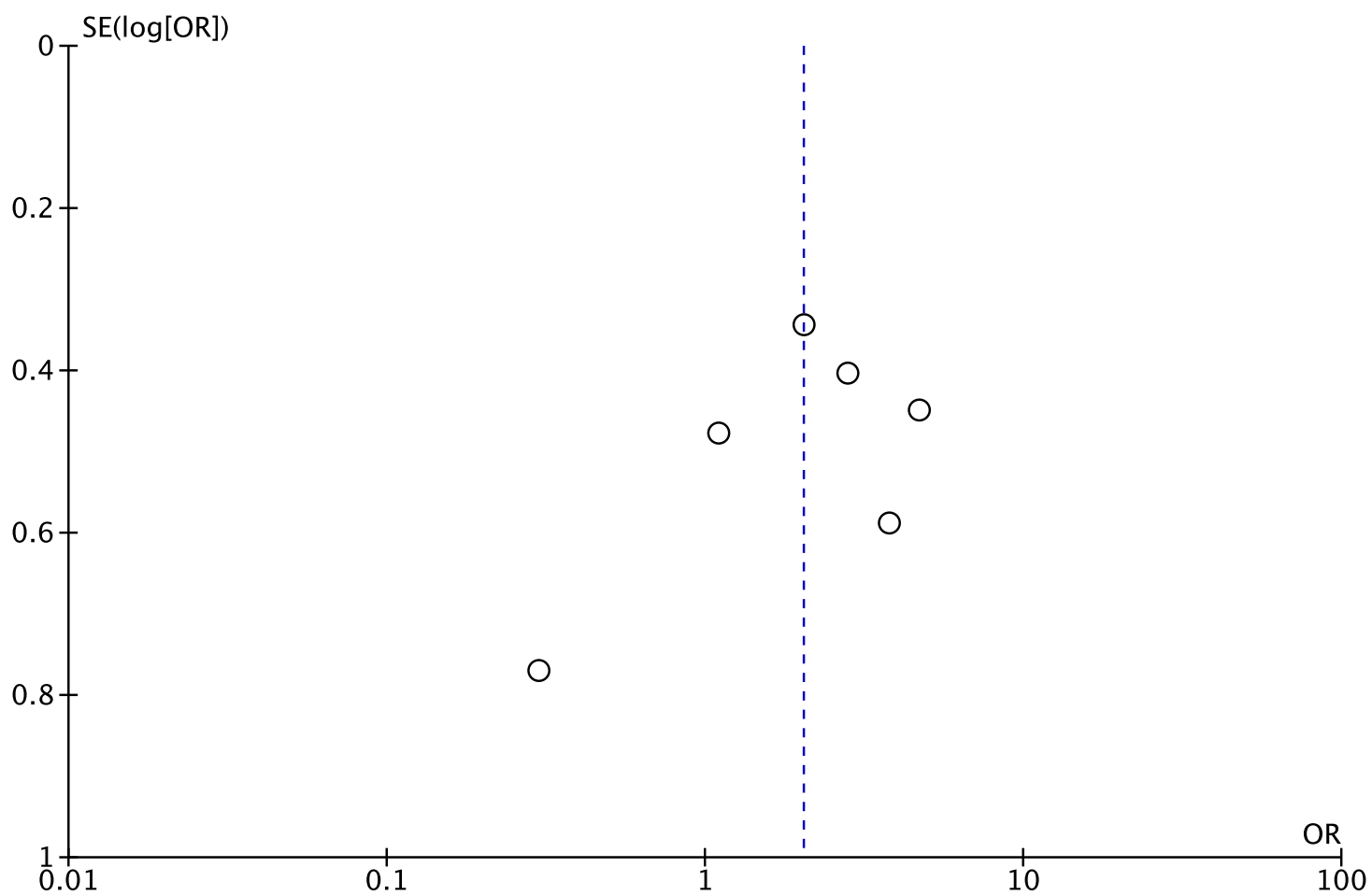

Supplement: Supplementary Figure 8 [file jiy444_suppl_supplementary_figure_8.pdf]

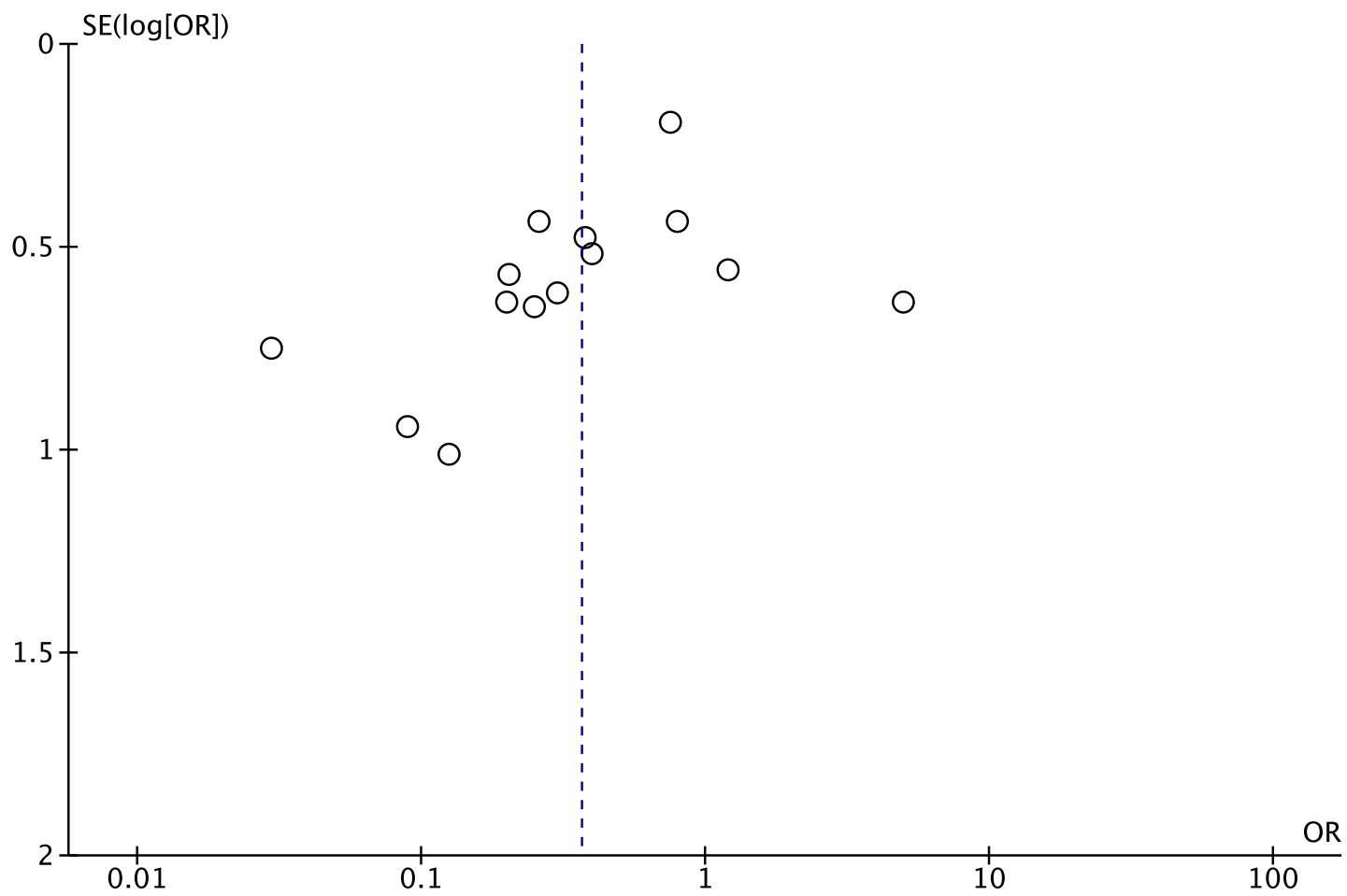

Supplement: Supplementary Figure 9 [file jiy444_suppl_supplementary_figure_9.pdf]

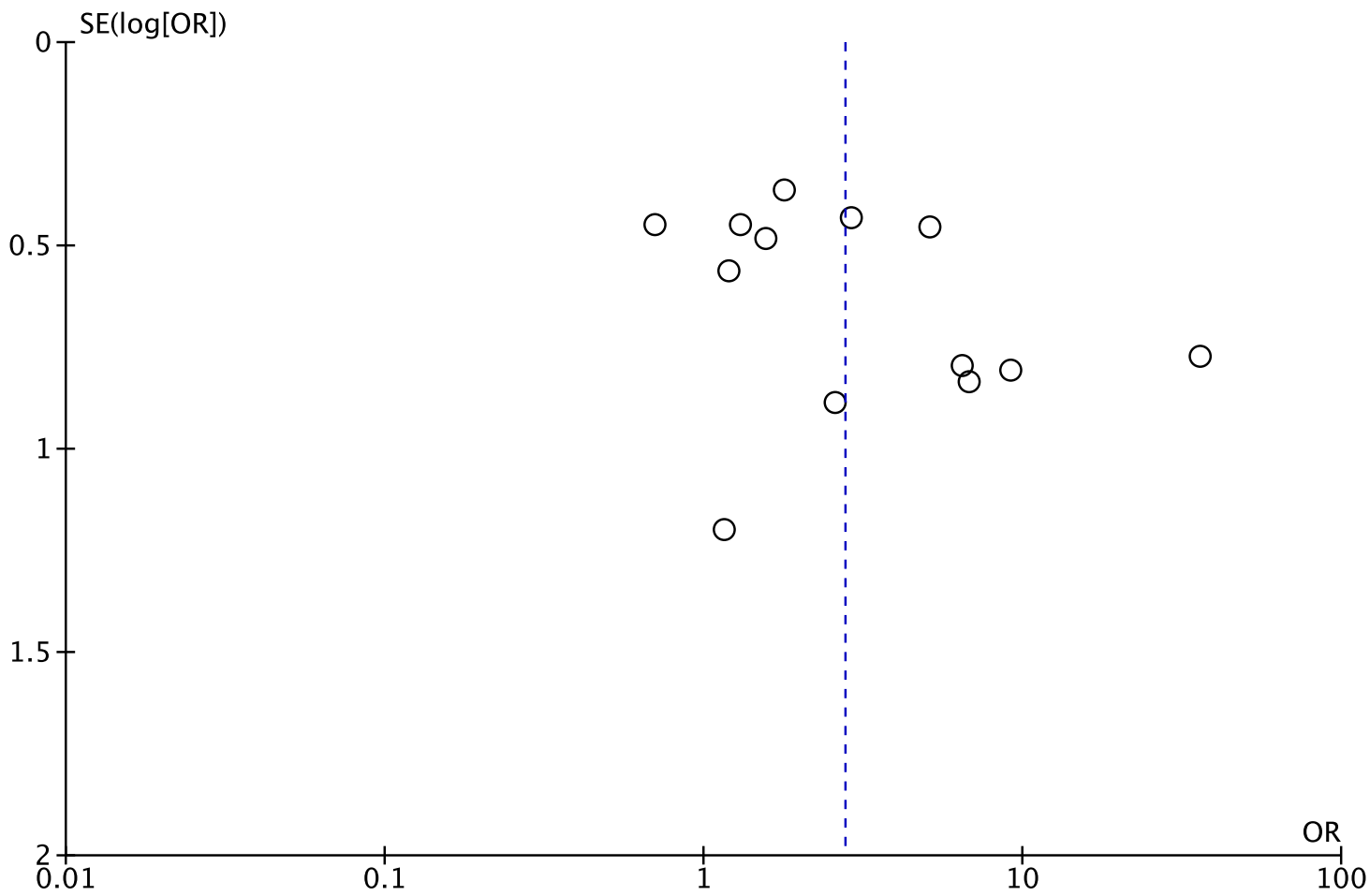

Supplement: Supplementary Figure 10 [file jiy444_suppl_supplementary_figure_10.pdf]

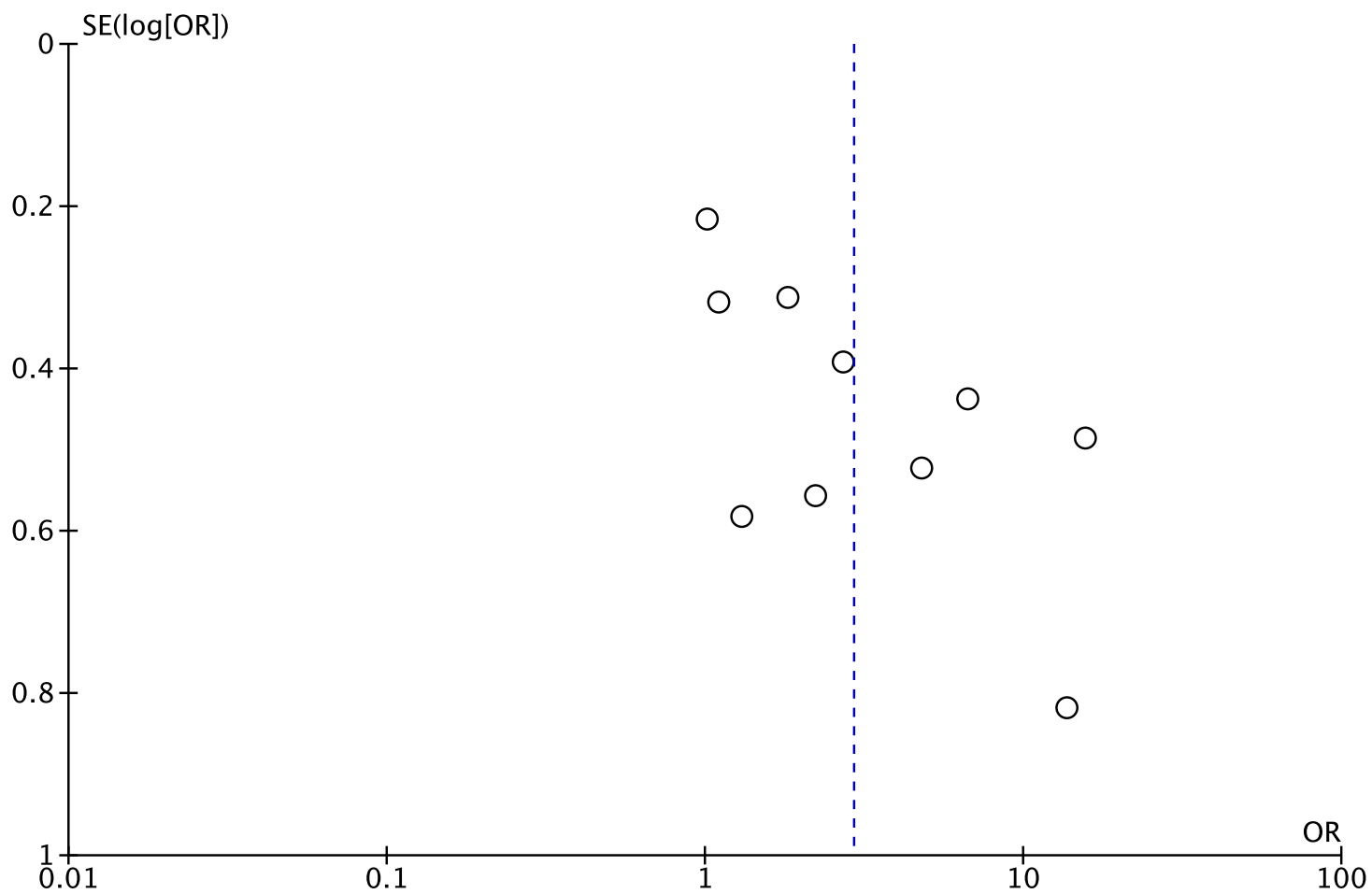

Supplement: Supplementary Figure 11 [file jiy444_suppl_supplementary_figure_11.pdf]

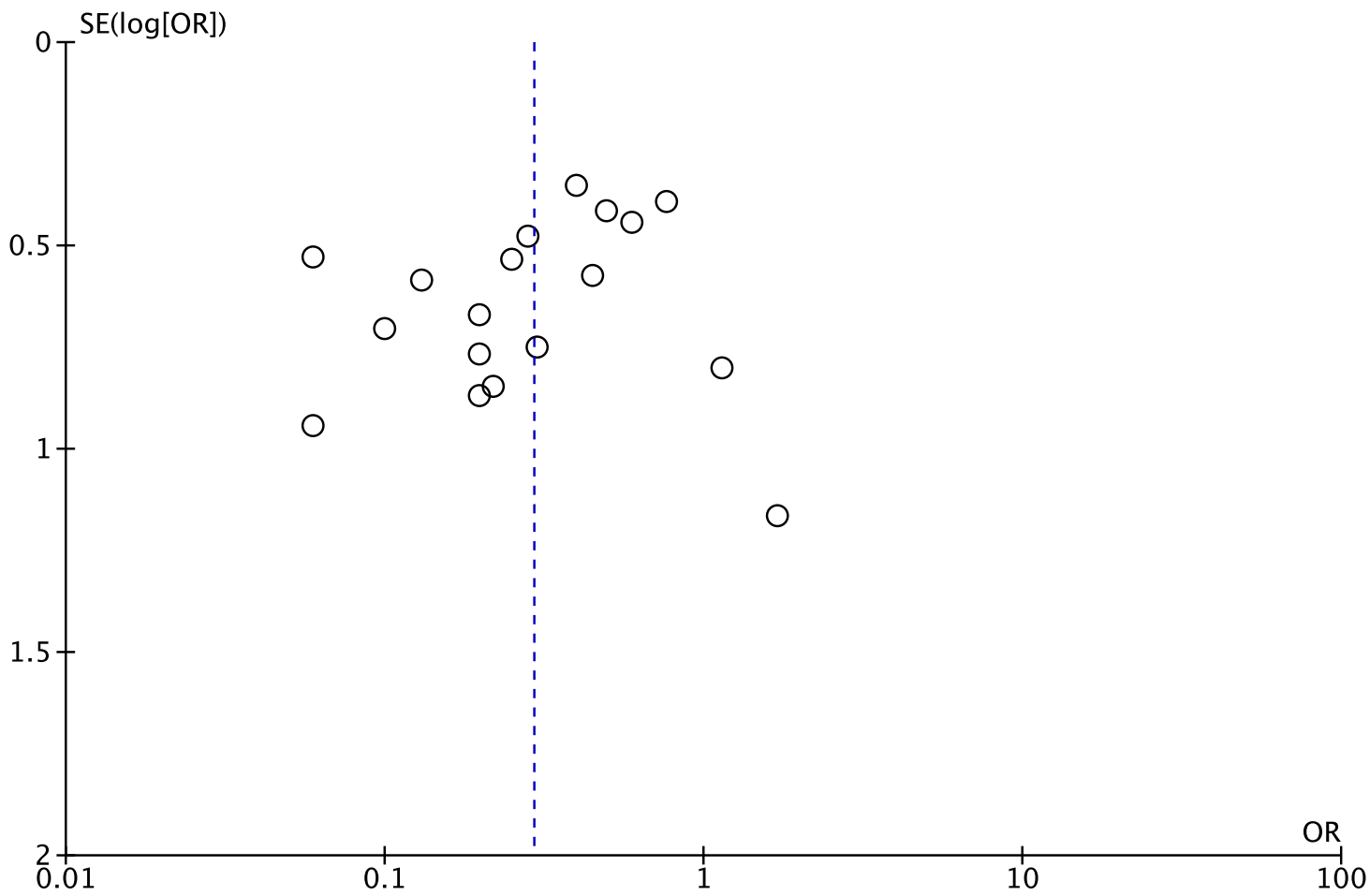

Supplement: Supplementary Figure 12 [file jiy444_suppl_supplementary_figure_12.pdf]

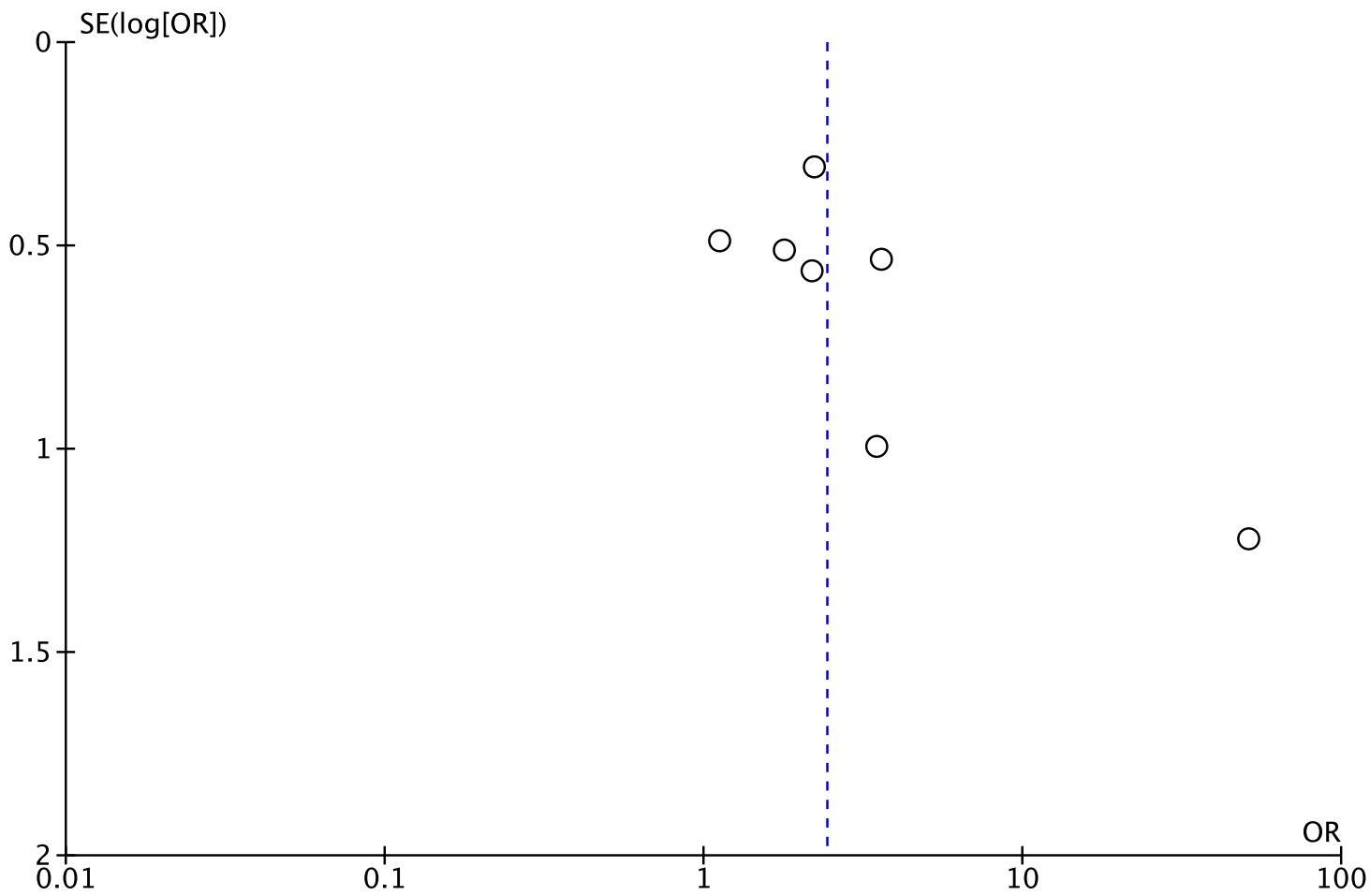

Supplement: Supplementary Figure 13 [file jiy444_suppl_supplementary_figure_13.pdf]

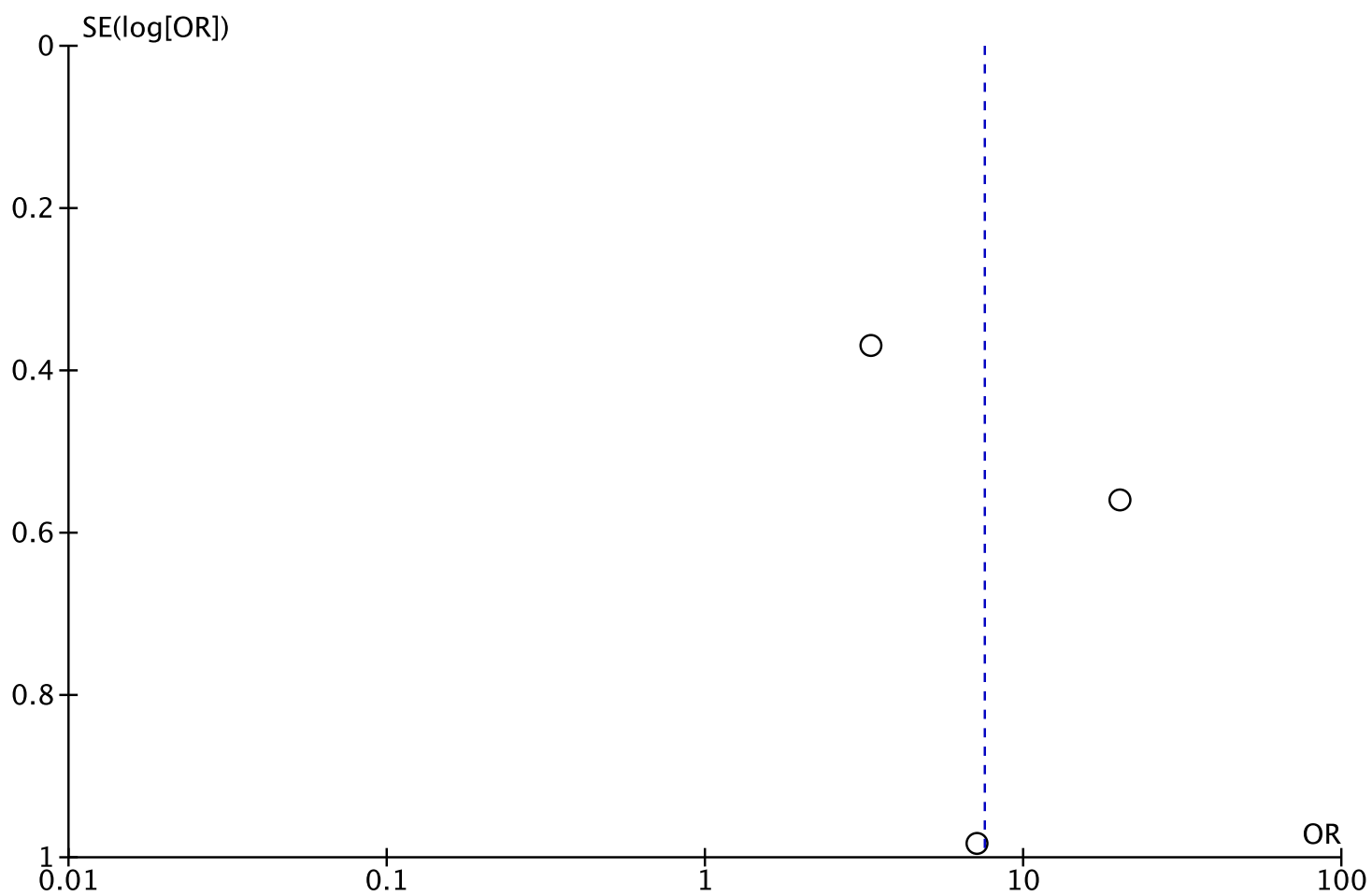

Supplement: Supplementary Figure 14 [file jiy444_suppl_supplementary_figure_14.pdf]
