# Supplementary material for: Individual and Household Risk Factors for Symptomatic Cholera Infection: A Systematic Review and Meta-analysis
Source: J Infect Dis. 2018 Aug 18;218(Suppl 3):S154–64. doi: 10.1093/infdis/jiy444 (PMC6188541; doi:10.1093/infdis/jiy444)
Supplement: Supplementary Material [file jiy444_suppl_supplementary_material.docx]

*Supplementary Figure 1.* Forest plot of studies included in meta-analysis assessing whether female gender was associated with risk of for symptomatic cholera. The summary odds ratio was calculated using random effects models. Heterogeneity is described using the Cochran’s Q test and the *I^2^* statistic.

*Supplementary Figure 2.* Forest plot of studies included in meta-analysis assessing whether exposure to street vendor food or food outside the household was associated with symptomatic cholera. The summary odds ratio was calculated using random effects models. Heterogeneity is described using the Cochran’s Q test and the *I^2^* statistic.

*Supplementary Figure 3.* Forest plot of studies included in meta-analysis assessing whether attending a large gathering or funeral was associated with symptomatic cholera. The summary odds ratio was calculated using random effects models. Heterogeneity is described using the Cochran’s Q test and the *I^2^* statistic.

*Supplementary Figure 4.* Forest plot of studies included in meta-analysis assessing whether use of an acidic food additive was associated with symptomatic cholera. The summary odds ratio was calculated using random effects models. Heterogeneity is described using the Cochran’s Q test and the *I^2^* statistic.

*Supplementary Figure 5.* Funnel plot to assess for publication bias for studies included in meta-analysis of association between gender and risk of symptomatic cholera.

*Supplementary Figure 6.* Funnel plot to assess for publication bias for studies included in meta-analysis of association between education and risk of symptomatic cholera.

*Supplementary Figure 7.* Funnel plot to assess for publication bias for studies included in meta-analysis of association between water source and risk of symptomatic cholera.

*Supplementary Figure 8.* Funnel plot to assess for publication bias for studies included in meta-analysis of association between water storage and risk of symptomatic cholera.

*Supplementary Figure 9.* Funnel plot to assess for publication bias for studies included in meta-analysis of association between water treatment and risk of symptomatic cholera.

*Supplementary Figure 10.* Funnel plot to assess for publication bias for studies included in meta-analysis of association between street vendor food or food outside the home and risk of symptomatic cholera.

*Supplementary Figure 11.* Funnel plot to assess for publication bias for studies included in meta-analysis of association between having a household contact with cholera and risk of symptomatic cholera.

*Supplementary Figure 12.* Funnel plot to assess for publication bias for studies included in meta-analysis of association between handwashing and risk of symptomatic cholera.

*Supplementary Figure 13.* Funnel plot to assess for publication bias for studies included in meta-analysis of association between attending a large gathering or funeral and risk of symptomatic cholera.

*Supplementary Figure 14.* Funnel plot to assess for publication bias for studies included in meta-analysis of association between use of an acidic food additive and risk of symptomatic cholera.

**Supplementary Table 1.** Data extracted from eligible studies.

| First Author |  |
| --- | --- |
| Year of Publication |  |
| Time Frame |  |
| Country |  |
| Setting | Urban/Rural |
|  | Hospital/Cholera Treatment Center/Clinic/Other |
| Study Design | Prospective/Retrospective |
|  | Cohort |
|  | Case-Control (matched / unmatched) |
|  | Other |
| Cholera case definition | Self-report |
|  | Clinical diagnosis |
|  | Medically-attended |
|  | Culture-positive |
|  | Other |
| Population | Description of cohort (if cohort study) |
|  | Description of case selection (if case-control study) |
|  | Number of Cases |
|  | Description of controls selection (if case-control study) |
|  | Number of Controls |
| Risk Factors | Demographic |
|  | Socioeconomic |
|  | Spatial Factor |
|  | Related to water source |
|  | Related to water treatment |
|  | Related to latrines |
|  | Household size |
|  | Household cholera contacts |
|  | Other household characteristics |
|  | Hand hygiene |
|  | Other hygiene |
|  | Related to food |
|  | Other assessed factors |
| Analysis | Did the study control for confounding beyond age, sex, and neighborhood? |
|  | Covariates controlled for |
| Bias Assessment^1^ | Selection |
|  | Comparability |
|  | Outcome |
| Additional Comments |  |

^1^Newcastle-Ottawa Scale

**Supplementary Table 2.** Summary of included studies

| **Study** | **Time Frame** | **Country** | **Study Design** | **Cholera Definition** | **Controls** | **Risk Factors Assessed** |
| --- | --- | --- | --- | --- | --- | --- |
| Acosta 2001 | 1997 | Tanzania | Matched Case-Control | Medically-attended cholera | Hospital | Socioeconomic  Spatial Factor^*^  Water  Latrine  Hygiene^*^  Food^*^ |
| Alam 1991 | 1990 | Bangladesh | Matched Case-Control | Culture-confirmed clinical cholera | Community | Ferritin |
| Ali 2011 | 1989-2003 | Bangladesh | Matched Case-control | Culture-confirmed clinical cholera | Community | Prior cholera infection |
| Anh 2011 | 2008 | Vietnam | Matched case control | Clinical cholera | Hospital | Demographics  Socioeconomic  Water  Food |
| Arifuzzaman 2011 | Not reported | Bangladesh | Matched case-control | Culture-confirmed clinical cholera | Household, Community | Lewis blood group |
| Baine 1974 | 1973 | Italy | Matched case control | Culture-confirmed clinical cholera | Household, Community | Food  Gastric surgery |
| Barua 1977 | 1976 | Philippines | Unmatched Case-control | Culture-confirmed clinical cholera | Non-cholera diarrhea | ABO Blood group |
| Beatty 2004 | 2000-2001 | Marshall Islands | Matched case-control | Medically-attended cholera; Culture-confirmed clinical cholera | Community | Socioeconomic  Water  Household size  Food  Cholera knowledge |
| Bhunia 2009 | 2006 | India | Matched case-control | Medically-attended cholera | Community | Socioeconomic  Water  Latrine  Household size  Cholera knowledge |
| Birmingham 1997 | 1992 | Burundi | Matched Case-Control | Medically-attended cholera | Community | Demographics  Spatial Factor  Water^*^  Hygiene^*^  Food |
| Biswas 2014 | 2012 | India | Matched case-control | Clinical cholera | Community | Water  Latrine  Cholera contact  Hygiene |
| Blake 1977 | 1974 | Portugal | 3 Matched case-control studies | Culture-confirmed clinical cholera | Community | Water  Food |
| Blake (2) 1977 | 1974 | Portugal | Matched Case-Control | Culture-confirmed clinical cholera | Community | Water |
| Blake 1980 | 1978 | USA | Matched Case-Control | Culture-confirmed clinical cholera | Community | Food |
| Boyce 1995 | 1994 | Thailand | Retrospective cohort | Culture-confirmed clinical cholera | Cruise ship passengers | Food |
| Burrowes 2017 | 2013-2014 | Bangladesh | Prospective cohort study nested within randomized controlled trial | Culture-confirmed clinical cholera | Household | Demographics  Water  Hygiene  Food  Travel/Gathering |
| Cardenas 1993 | 1992 | Colombia | Cross-sectional Survey / Case-control | Positive history and vibriocidal titers (cross-sectional) /Culture-confirmed clinical cholera (case-control study) | Community | Demographics^*^  Socioeconomic  Water^*^  Latrine  Cholera contact  Food |
| CDC 2009 | 2007 | South Sudan | Matched case-control | Medically-attended cholera | Community | Demographics  Spatial Factor  Water  Latrine  Hygiene  Food^*^  Travel/Gathering |
| Chaudhuri 1978 | 1977 | India | Unmatched case-control | Culture-confirmed clinical cholera | Clinic | Blood group saliva secretors |
| Clemens 1989 | 1985 | Bangladesh | Secondary analysis of RCT of vaccine (Retrospective cohort of placebo recipients) | Culture-confirmed clinical cholera | Community | ABO Blood group |
| Clemens 1990 | 1985-1986 | Bangladesh | Matched case-control study performed during cholera vaccine trial | Culture-confirmed severe cholera (1- death during hospitalization or 2-signs of significant dehydration or depressed mental status) | Community | Spatial Factor*  Water*  Breastfeeding* |
| Clemens 1995 | 1985 | Bangladesh | Secondary analysis of RCT (control arm only) - matched case-control | Culture-confirmed clinical cholera | Community | *H. pylori* IgG^*^ |
| Colombara 2013 | 2000-2008 | Bangladesh | Unmatched case-control | Culture-confirmed clinical cholera | Non-cholera diarrhea | Demographics^*^  Socioeconomic^*^  Spatial Factor  Water  Latrine  Cholera contact^*^  Breastfeeding^*^  Vitamin A^*^  Malnutrition |
| Colombara 2014 | 2000-2008 | Bangladesh | Unmatched case-control | Culture-confirmed clinical cholera | Shigella | Demographics^*^  Socioeconomic^*^  Spatial Factor  Water  Latrine  Household size  Cholera contact^*^ |
| Conroy 2001 | 1997-1998 | Kenya | Secondary analysis of RCT | Clinical cholera | Community | Water |
| Cummings 2012 | 2010 | Uganda | Unmatched case-control | Medically-attended cholera | Community | Demographics^*^  Water^*^  Latrine^*^  Cholera contact^*^  Hygiene^*^  Food^*^ |
| Datta 2012 | 2010 | India | Retrospective cohort | Medically-attended cholera | Community | Water^*^  Cognitive function^*^ |
| De Guzman 2015 | 2012 | Philippines | Matched case-control | Medically-attended cholera | Household, community | Demographics^*^  Spatial Factor^*^  Water^*^  Latrine^*^  Hygiene^*^ |
| Debes 2016 | 1991-2000 | Bangladesh | Matched case-control | Culture-confirmed clinical cholera | Community | Spatial Factor^*^ |
| Deepthi 2013 | 2010 | India | Unmatched case-control | Culture-confirmed clinical cholera | Community | Socioeconomic  Water  Latrine  Hygiene |
| DuBois 2006 | 2003-2004 | Zambia | Matched case-control | Medically-attended cholera | Community | Water^*^  Latrine  Hygiene^*^  Food^*^ |
| Dunkle 2011 | 2010 | Haiti | Matched Case-Control | Medically-attended cholera | Community | Socioeconomic  Water^*^  Latrine  Hygiene^*^  Food^*^ |
| Eberhart-Phillips 1996 | 1992 | Argentina, Peru, United States | Unmatched case-control | Culture-confirmed clinical cholera | Flight passengers | Water^*^  Food^*^ |
| Emch 1999 | 1992-1994 | Bangladesh | Unmatched case-control | Culture-confirmed clinical cholera | Community | Demographics  Socioeconomic  Spatial Factor  Water  Latrine  Household size  Food |
| Evans 1997 | Not reported | Bangladesh | Unmatched case-control | Culture-confirmed clinical cholera | Non-cholera diarrhea | Gastric acidity |
| Fatiregun 2013 | 2012 | Nigeria | Matched Case-Control | Clinical cholera | Community | Cholera contact |
| Finelli 1992 | 1991 | USA | Unmatched case-control | Culture-confirmed clinical cholera | People present at meal | Food |
| Fukuda 1995 | 1991 | Peru | Unmatched case-control | Culture-confirmed clinical cholera | Non-cholera diarrhea | Demographics  Water  Cholera contact  Food |
| Glass 1985 | 1979-1982 | Bangladesh | 2 unmatched case-control studies | Culture-confirmed clinical cholera | Clinic, family contacts, blood donors | ABO Blood group |
| Grandesso 2014 | 2011 | Haiti | 2 matched case-control studies | Microbiologically-confirmed clinical cholera (rapid test) | Community | Socioeconomic^*^  Water^*^  Latrine^*^  Household size^*^  Cholera contact  Hygiene  Food^*^  Cholera knowledge^*^ |
| Gunn 1979 | 1978 | Bahrain | Matched case-control | Culture-confirmed clinical cholera | Household | Water  Hygiene  Breastfeeding  Food |
| Gunnlaugsson 1998 | 1994 | Guinea-Bissau | Unmatched case-control | Medically-attended cholera | Community | Water  Travel/Gathering  Cholera contact |
| Harris 2005 | 2001-2004 | Bangladesh | Prospective cohort | Microbiologically-confirmed cholera (culture or rise in vibriocidal antibody) | Household | ABO Blood Group^*^  Baseline vibriocidal titer^*^ |
| Harris 2008 | 2001-2006 | Bangladesh | Prospective cohort | Microbiologically-confirmed cholera (culture or rise in vibriocidal antibody) | Household | Zinc, Vitamin A  Baseline vibriocidal titer  LPS-specific antibodies  Height/Weight-fod-age  ABO blood group |
| Hatch 1994 | 1988 | Malawi | Unmatched case-control | Medically-attended cholera | Community | Demographics^*^  Socioeconomic^*^  Water^*^  Latrine  Household size^*^  Hygiene^*^  Food |
| Haus-Cheymol 2012 | 2010 | Haiti | Retrospective cohort study | Clinical cholera | Community | Food |
| Hoge 1996 | 1993-1994 | Thailand | Unmatched case-control | Culture-confirmed clinical cholera | Hospital | Socioeconomic^*^  Water^*^  Cholera contact^*^  Hygiene^*^  Food^*^  Travel/Gathering^*^ |
| Holmberg 1984 | 1982 | Micronesia | Matched case-control | Culture-confirmed clinical cholera | Community | Cholera contact |
| Hornick 1971 | 1971 | USA | Experimental innoculation of volunteers | Culture-confirmed clinical cholera | Volunteer | Gastric acidity |
| Hughes 1982 | 1973-1874 | Bangladesh | Prospective cohort (case-control for comparison included) | Culture-confirmed clinical cholera | Non-cholera diarrhea | Water  Hygiene |
| Hutin 2003 | 1995-1996 | Nigeria | Unmatched case-control | Clinical cholera | Hospital | Water^*^  Hygiene^*^  Food |
| Ishaku 2014 | 2013 | Nigeria | Unmatched case-control | Clinical cholera | Community | Water  Latrine  Cholera contact  Hygiene  Food |
| Karlsson 2013 | 2004-2005 | Bangladesh | Cohort / unmatched case-control | Culture-confirmed clinical cholera | Community | Genetic |
| Killewo 1989 | 1986 | Tanzania | Matched case-control | Culture-confirmed clinical cholera | Community | Water  Cholera contact  Food  Travel/Gathering |
| Kirk 2005 | 2000 | Federated States of Micronesia | Matched case-control | Clinical cholera | Community | Water^*^  Latrine  Hygiene |
| Kone-Coulibaly 2010 | 2008-2009 | Zimbabwe | Unmatched case-control | Clinical cholera | Community | Socioeconomic^*^  Water  Cholera contact^*^  Hygiene  Food^*^  Travel/Gathering |
| Koo 1996 | 1993 | Guatemala | Matched case-control | Clinical cholera | Community | Water  Food^*^ |
| Levine 1981 | 1981 | USA | Experimental innoculation of volunteers | Culture-confirmed clinical cholera | Volunteers not previously infected with cholera | Prior infection |
| Lim-Quizon 1994 | 1989 | Philippines | Unmatched case-control | Culture-confirmed clinical cholera | Hospital | Water^*^  Food^*^ |
| Lowry 1989 | 1986 | USA | 3 matched case-control studies (analyzed unmatched) | Clinical cholera | Community | Food  Gastric surgery  Antacids |
| Lucas 2005 | 2003-2004 | Mozambique | Secondary analysis of RCT (matched case-control study) | Culture-confirmed clinical cholera | Community | Demographics  Socioeconomic  Water  Latrine  Household size  Food |
| Luquero 2011 | 2008 | Guinea-Bissau | Cluster analysis among cohort of househodls | Clinical cholera | Community | Spatial Factor |
| Mahamud 2012 | 2009 | Kenya | Matched case-control | Clinical cholera | Community | Demographics  Water^*^  Latrine  Cholera contact  Hygiene^*^  Food |
| Matias 2017 | 2012-2014 | Haiti | Matched case-control | Culture-confirmed clinical cholera | Community | Demographics^*^  Socioeconomic  Water^*^  Latrine^*^  Household size  Cholera contact  Hygiene  Food  Antacids  Cholera knowledge |
| McIntyre 1979 | 1977 | Gilbert Islands | 2 matched case-control studies | clinical cholera (Study 1) / Culture-confirmed clinical cholera (study 2) | Community | Water  Food |
| Moradi 2016 | 2011 | Iran | Matched case-control | Culture-confirmed clinical cholera | Community | Cholera contact  Food^*^  Travel/Gathering |
| Moren 1991 | 1988 | Malawi | Matched case-control | Clinical cholera | Community | Water  Food  Travel/Gathering |
| Mosley 1968 | 1965-1966 | East Pakistan | Prospective cohort | Culture-confirmed clinical cholera | Household | Baseline vibriocidal antibody titer |
| Mridha 2011 | 2010 | India | Unmatched case-control | Clinical cholera | Community | Water^*^  Hygiene^*^  Food^*^ |
| Mugoya 2008 | 2005 | Kenya | Two matched case-control studies | Culture-confirmed clinical cholera | Community | Water^*^  Latrine^*^  Hygiene^*^  Food^*^  Travel/Gathering^*^ |
| Mujica 1994 | 1991 | Peru | Matched case-control | Clinical cholera | Community | Water^*^  Food^*^ |
| Mukherjee 2011 | 2004-2008 | India | 4 case-control studies, one retrospective cohort | Clinical cholera | Community | Water  Hygiene |
| Nguyen 2014 | 2012 | Sierra Leone | Matched case-control | Clinical cholera | Community | Socioeconomic^*^  Water^*^  Cholera contact  Food^*^  Travel/Gathering |
| Nguyen 2017 | 2010 | Vietnam | Matched case-control | Culture-confirmed clinical cholera | Community | Demographics^*^  Socioeconomic^*^  Water^*^  Latrine  Cholera contact^*^  Hygiene^*^  Food^*^  Travel/Gathering^*^ |
| Noorhaida 2010 | 2007 | Malaysia | Unmatched case-control | Culture-confirmed clinical cholera | Household, communitry | Food |
| Nsagha 2015 | 2011 | Cameroon | Unmatched case-control | Clinical cholera | Community | Demographics  Socioeconomic  Water  Latrine  Food |
| O'Connor 2011 | 2010 | Haiti | Matched case-control | Clinical cholera | Community | Socioeconomic^*^  Water^*^  Latrine^*^  Hygiene  Food^*^ |
| Opare 2012 | 2010 | Ghana | Unmatched case-control | Clinical cholera | Community | Water  Latrine  Cholera contact  Hygiene  Food |
| Quick 1995 | 1991 | El Salvador | Matched case-control | Culture-confirmed clinical cholera | Community | Water  Hygiene  Food  Cholera knowledge |
| Qureshi 2006 | 1994 | Guinea-Bissau | Prospective cohort | Clinical cholera (with vibriocidal titer equal or greater than 20) | Community | Breastfeeding^*^ |
| Rahman 2009 | 1992 | Bangladesh | Family-matched case control | Culture-confirmed clinical cholera | Family, community | Demographics^*^  Socioeconomic^*^  Latrine^*^  Sibling of cholera case^*^ |
| Reller 2001 | 2001 | Madagascar | Matched case-control | Clinical cholera | Community | Water^*^  Hygiene  Food^*^ |
| Ries 1992 | 1991 | Peru | 2 Matched case-control studies | Clinical cholera | Community | Water^*^  Food^*^ |
| Riley 1987 | 1986 | Bangladesh | Case-control | Clinical cholera / culture-confirmed cholera | Community | Breastfeeding |
| Rodrigues 1997 | 1994 | Guinea-Bissau | Matched case-control | Clinical cholera | Community | Water^*^  Latrine  Hygiene^*^  Food^*^  Acidic food additive^*^ |
| Rodrigues 2000 | 1996 | Guinea-Bissau | Matched case-control | Clinical cholera | Community | Water^*^  Food^*^  Acidic food additive^*^ |
| Rosewell 2011 | 2009 | Papua New Guinea | Matched case-control | Clinical cholera | Community | Water  Cholera contact^*^  Hygiene  Food  Travel/Gathering^*^ |
| Rosewell 2012 | 2010 | Papua New Guinea | Unmatched case-control | Clinical cholera | Hospital | Demographics^*^  Water  Latrine^*^  Cholera contact^*^  Hygiene^*^  Food  Travel/Gathering |
| Ryder 1986 | 1974-1975 | Bangladesh | Unmatched case-control | Culture-confirmed clinical cholera | Non-cholera diarrhea | Receiving IV fluids |
| Sack 1972 | 1969 | Bangladesh | Prospective cohort | Culture-confirmed clinical cholera | Community | Gastric acid production |
| Saha 2004 | 2001-2002 | Bangladesh | Prospective cohort | Culture-confirmed clinical cholera (or clinical cholera with 4-fold increase in vibriocidal titer) | Household | Cholera contact  Baseline vibriocidal titers |
| Saha 2017 | 2011-2013 | Bangladesh | Unmatched case-control study within prospective cohort | Culture-confirmed clinical cholera (or clinical cholera with 4-fold increase in vibriocidal titer) | Community | Demographics^*^  Socioeconomic^*^  Spatial Factor^*^  Water  Latrine  Household size  Population density^*^ |
| Sasaki 2008 | 2003-2004 | Zambia | Matched case-control | Clinical cholera | Community | Water  Latrine  Hygiene |
| Seas 2000 | 1995 | Peru | Unmatched case-control | Culture-confirmed clinical cholera | Non-cholera diarrhea | Water  Latrine  Food |
| Sema Baltazar 2017 | 2011-2015 | Mozambique | Unmatched case-control | Culture-confirmed clinical cholera | Non-cholera diarrhea | Demographics^*^  Water^*^  Year^*^  HIV^*^ |
| Shapiro 1999 | 1997-1998 | Kenya | Matched case-control | Clinical cholera | Clinic | Water^*^  Latrine^*^  Food^*^  Travel/Gathering^*^ |
| Shultz 2009 | 2005 | Kenya | Matched Case-Control | Clinical cholera | Community | Demographics^*^  Water^*^  Latrine^*^  Hygiene  Food |
| Siddiqui 2006 | 2002-2003 | Pakistan | Two matched case-control studies during two outbreaks | Culture-confirmed clinical cholera | Community | Water  Latrine  Cholera contact  Travel/Gathering |
| Sinclair 1982 | 1981 | South Africa | Matched case-control | Culture-confirmed clinical cholera | Community | Water |
| St Louis 1990 | 1986 | Guinea-Bissau | Matched case-control / case-control (funeral) | Clinical cholera | Community | Water  Hygiene^*^  Food^*^  Acidic food additive^*^ |
| Sur 2005 | 2003-2004 | India | Prospective cohort study | Culture-confirmed clinical cholera | Community | Demographics  Socioeconomic^*^  Water^*^  Cholera contact^*^  Hygiene^*^ |
| Swaddiwudhip 1989 | 1984 | Thailand | Matched Case-Control | Culture-confirmed clinical cholera | Hospital | Food  Antacid |
| Swerdlow 1992 | 1991 | Peru | Matched Case-Control | Culture-confirmed clinical cholera | Community | Water^*^  Food  Travel/Gathering^*^ |
| Swerdlow 1997 | 1990 | Malawi | Two case-control studies (matched individuals / unmatched households) | Clinical cholera | Community | Water  Socioeconomic  Hygiene  Food |
| Tauxe 1988 | 1984 | Mali | 2 Matched-case control studies (one per village 1 and 4) | Clinical cholera | Community | Water  Food |
| Ujjiga 2015 | 2014 | South Sudan | Matched case-control | Clinical cholera | Community | Water  Food^*^  Travel/Gathering^*^ |
| Uthappa 2015 | 2013 | India | Matched case-control | Clinical cholera | Community | Demographics  Socioeconomic  Water^*^  Household size^*^  Hygiene^*^  Travel/Gathering |
| Van Loon 1990 | Not reported | Bangladesh | Matched case-control | Culture-confirmed clinical cholera | Household | Gastric acid production |
| Von Seidlein 2008 | 2005-2006 | Mozambique | Matched case-control | Culture-confirmed clinical cholera | Community | Demographics^*^  Socioeconomic^*^  Water^*^  Latrine^*^  Household size^*^  Food^*^  HIV^*^ |
| Weber 1994 | 1991 | Ecuador | Matched case-control | Clinical cholera | Community | Water^*^  Hygiene  Food^*^ |

^*^ Included in multivariable analysis

**Supplementary Table 3.** Bias within included studies

|  | Bias^1^ | | |
| --- | --- | --- | --- |
|  | Selection^2^ | Comparability^3^ | Outcome^4^ |
| Acosta 2001 | *** | ** | * |
| Alam 1991 | *** |  | *** |
| Ali 2011 | **** | ** | *** |
| Anh 2011 | ** |  | ** |
| Arifuzzaman 2011 | *** |  | ** |
| Baine 1974 | ** |  | ** |
| Barua 1977 | * |  | *** |
| Beatty 2004 | *** |  | * |
| Bhunia 2009 | *** |  | * |
| Birmingham 1997 | *** |  | * |
| Biswas 2014 | *** |  | * |
| Blake 1977 | ** (* for Lisbon) |  | ** |
| Blake (2) 1977 | *** |  | ** |
| Blake 1980 | *** |  | ** |
| Boyce 1995 | *** |  | ** |
| Burrowes 2017 | **** |  | ** |
| Cardenas 1993 | *** | ** | ** |
| CDC 2009 | *** | ** | * |
| Chaudhuri 1978 | * |  | ** |
| Clemens 1989 | **** |  | ** |
| Clemens 1990 | *** | ** | ** |
| Clemens 1995 | *** | ** | *** |
| Colombara 2013 | ** | ** | * |
| Colombara 2014 | ** | ** | ** |
| Conroy 2001 | **** |  | ** |
| Cummings 2012 | *** | ** | * |
| Datta 2012 | *** |  | * |
| De Guzman 2015 | ** | ** | ** |
| Debes 2016 | *** | ** | *** |
| Deepthi 2013 | *** |  | ** |
| DuBois 2006 | *** | ** | * |
| Dunkle 2011 | *** | ** | * |
| Eberhart-Phillips 1996 | *** | ** | * |
| Emch 1999 | ** |  | ** |
| Evans 1997 | * |  | *** |
| Fatiregun 2013 | *** |  | ** |
| Finelli 1992 | * |  | *** |
| Fukuda 1995 | ** |  | * |
| Glass 1985 | *** |  | ** |
| Grandesso 2014 | *** | ** | * |
| Gunn 1979 | ** |  | * |
| Gunnlaugsson 1998 | *** |  | ** |
| Harris 2005 | *** | ** | *** |
| Harris 2008 | **** |  | *** |
| Hatch 1994 | *** | ** | * |
| Haus-Cheymol 2012 | **** |  | *** |
| Hoge 1996 | ** | ** | * |
| Holmberg 1984 | ** |  | * |
| Hornick 1971 | n/a | n/a | n/a |
| Hughes 1982 | * |  | ** |
| Hutin 2003 | * | * | * |
| Ishaku 2014 | ** |  | * |
| Karlsson 2013 | n/a | n/a | n/a |
| Killewo 1989 | *** |  | * |
| Kirk 2005 | *** | ** | * |
| Kone-Coulibaly 2010 | ** | ** | * |
| Koo 1996 | ** | * | * |
| Levine 1981 | n/a | n/a | n/a |
| Lim-Quizon 1994 | * | ** | * |
| Lowry 1989 | *** |  | ** |
| Lucas 2005 | **** |  | *** |
| Luquero 2011 | **** |  | ** |
| Mahamud 2012 | ** | ** | ** |
| Matias 2017 | ** | ** | ** |
| McIntyre 1979 | ** |  | * |
| Moradi 2016 | * | ** | ** |
| Moren 1991 | *** |  | * |
| Mosley 1968 | **** |  | *** |
| Mridha 2011 | ** | ** | ** |
| Mugoya 2008 | ** | ** | ** |
| Mujica 1994 | ** | ** | ** |
| Mukherjee 2011 | * |  | * |
| Nguyen 2014 | *** | ** | ** |
| Nguyen 2017 | **** | ** | ** |
| Noorhaida 2010 | *** |  | * |
| Nsagha 2015 | ** |  | ** |
| O'Connor 2011 | *** | ** | ** |
| Opare 2012 | ** |  | ** |
| Quick 1995 | ** |  | * |
| Qureshi 2006 | **** | * | *** |
| Rahman 2009 | *** | ** | ** |
| Reller 2001 | ** | * | * |
| Ries 1992 | *** | ** | ** |
| Riley 1987 | * |  | * |
| Rodrigues 1997 | *** | ** | * |
| Rodrigues 2000 | *** | ** | ** |
| Rosewell 2011 | *** | ** | ** |
| Rosewell 2012 | ** | ** | ** |
| Ryder 1986 | ** |  | *** |
| Sack 1972 | ** |  | *** |
| Saha 2004 | **** |  | *** |
| Saha 2017 | ** | ** | *** |
| Sasaki 2008 | *** |  | ** |
| Seas 2000 | ** |  | ** |
| Sema Baltazar 2017 | * | ** | *** |
| Shapiro 1999 | * | ** | ** |
| Shultz 2009 | ** | ** | ** |
| Siddiqui 2006 | ** |  | ** |
| Sinclair 1982 | ** |  | * |
| St Louis 1990 | *** | ** | ** |
| Sur 2005 | *** | * | *** |
| Swaddiwudhip 1989 | ** |  | ** |
| Swerdlow 1992 | *** | * | ** |
| Swerdlow 1997 | *** |  | ** |
| Tauxe 1988 | *** |  | ** |
| Ujjiga 2015 | *** | ** | ** |
| Uthappa 2015 | *** | ** | ** |
| Van Loon 1990 | ** |  | *** |
| Von Seidlein 2008 | ** | ** | * |
| Weber 1994 | ** | ** | * |

^1^Newcastle-Ottawa Scale[9]

^2^A study could be awarded a maximum of four stars in this category

^3^A study could be awarded a maximum of two stars in this category

^4^A study could be awarded a maximum of three stars in this category
